# Burnout and the Brain—A Mechanistic Review of Magnetic Resonance Imaging (MRI) Studies

**DOI:** 10.3390/ijms26178379

**Published:** 2025-08-28

**Authors:** James Chmiel, Donata Kurpas

**Affiliations:** 1 Institute for Neurofeedback and tDCS, 3 Maja 27, 70-215 Szczecin, Poland; 2 Faculty of Health Sciences, Department of Family and Pediatric Nursing, Wrocław Medical University, 51-618 Wrocław, Poland

**Keywords:** burnout, magnetic resonance imaging, neural correlates, exhaustion, fMRI, MRI

## Abstract

Occupational burnout is ubiquitous yet still debated as a disease entity. Previous reviews surveyed multiple biomarkers but left their neural substrate unclear. We therefore asked: What, if any, reproducible magnetic-resonance signature characterises burnout? Following PRISMA principles adapted for mechanistic synthesis, two reviewers searched PubMed, Scopus, Google Scholar, ResearchGate and Cochrane from January 2000 to May 2025 using “MRI/fMRI” AND “burnout”. After duplicate removal and multi-stage screening, 17 clinical studies met predefined inclusion criteria (English language, MRI outcomes, validated burnout diagnosis). In total, ≈1365 participants were scanned, 880 with clinically significant burnout and 470 controls. Uniform Maslach Burnout Inventory thresholds defined cases; most studies matched age and sex, and all excluded primary neurological disease. Structural morphometry (8/17 studies) revealed consistent amygdala enlargement—predominantly in women—and grey-matter loss in dorsolateral/ventromedial prefrontal cortex and striatal caudate–putamen, while hippocampal volume remained unaffected, distinguishing burnout from PTSD or depression. Resting-state and task fMRI (9/17 studies) showed fronto-cortical hyper-activation, weakened amygdala–ACC coupling, and progressive fragmentation of rich-club networks, collectively indicating compensatory executive overdrive and global inefficiency. Two longitudinal cohorts and several intervention sub-studies demonstrated partial reversal of cortical thinning and limbic hyper-reactivity after mindfulness, exercise, cognitive-behavioural therapy, neurofeedback, or rTMS, underscoring plasticity. Across heterogeneous paradigms and populations, MRI converges on a coherent, sex-modulated but reversible brain-networkopathy that satisfies objective disease criteria. These findings justify early neuro-imaging-based triage, circuit-targeted therapy, and formal nosological recognition of burnout as a mental disorder, with policy ramifications for occupational health and insurance parity.

## 1. Introduction

The World Health Organization formally recognises burnout as an occupational phenomenon rather than a mental disorder [1]. ICD-11 describes it as “a syndrome conceptualised as resulting from chronic workplace stress that has not been successfully managed” [1]. Burnout manifests across three diagnostic axes—emotional exhaustion, cynicism (depersonalisation), and reduced professional efficacy—formalised in the Maslach Burnout Inventory and the ICD-11 definition and reaffirmed by recent reviews [2,3]. Emotional exhaustion is typically the earliest and most salient feature, with workers reporting unrelenting fatigue and feeling “drained” even after rest, a pattern observed in nearly half of frontline clinicians in a 2024 Czech survey [4]. Cognitive drag quickly follows lapses in concentration, memory slips, and slower decision-making were the strongest predictors of a high-risk burnout profile [5]. Affective changes emerge as irritability, pessimism, and defensive emotional distancing or cynicism toward clients and colleagues, a coping stance that temporarily blunts stress yet erodes empathy and job satisfaction [6]. Physical manifestations are equally common: a study from 2023 catalogued headaches, gastrointestinal upset, and sleep disturbance as frequent correlates of burnout among the general population, reflecting spill-over of chronic stress into the body [7].

The size of the burnout problem varies widely by occupation, region, and the instrument used, but the overall picture is one of high—and often increasing—prevalence. In physicians, a landmark JAMA systematic review of 182 studies reported point-estimates ranging from 0% to 80%, with a median of roughly 67% of doctors meeting burnout criteria at any given time [8]. Nurses show a similarly worrying picture: an umbrella review of 14 meta-analyses concluded that one-third experience high emotional exhaustion, one-quarter report pronounced depersonalization, and another one-third feel a low sense of professional accomplishment [9]. Outside clinical settings, a 2024 synthesis of surveys in the public-health workforce calculated a pooled prevalence of 39% [10]. Data from non-Western settings are equally striking; for example, an extensive cross-sectional study of 4338 salaried employees in Southeast Asian countries found that 62.9% met the cut-off for burnout on the Maslach Burnout Inventory [11]. Although absolute figures differ because of heterogeneous cut-offs and cultural response styles, the convergence of these large datasets confirms that burnout is endemic wherever high emotional labour, excessive workload, and low autonomy intersect in modern workplaces.

Burnout carries heavy organisational costs. A 2025 analysis estimated that disengagement and burnout cost U.S. employers 0.2–2.9 × the average annual health-insurance premium and 3.3–17.1 × annual training costs per employee [12]. In healthcare, a 2022 BMJ meta-analysis found burnout doubled physicians’ intention to leave, increased actual turnover, and was linked to more patient-safety incidents and lower professionalism [13].

Burnout exerts a broad and well-documented impact on both mental and physical health. Mixed-methods and syntheses consistently show that individuals with high burnout scores are far more likely to screen positive for major depression and generalized anxiety, and they report suicidal thoughts at rates up to twice those of their non-burned out counterparts [14]. The syndrome’s reach, however, extends well beyond psychological morbidity. A 2024 systematic review found that chronically burned-out employees had a significantly higher risk of incident cardiovascular disease, including myocardial infarction and stroke [15]. Parallel evidence links burnout (or the closely related construct of vital exhaustion) to dysregulation of metabolic pathways: pooled analyses indicate positive associations with central obesity, insulin resistance, and the composite metabolic syndrome, predisposing to type 2 diabetes [16,17]. Crucially, these adverse consequences appear to translate into early mortality; in a 10-year Finnish cohort, each unit increase in baseline burnout score predicted a 35% rise in all-cause mortality among workers younger than 45 years [18]. Together, these findings underscore that burnout is not merely a transient mood disturbance but a multidimensional threat to long-term health, amplifying the burden of both psychiatric and cardiometabolic disease across working populations.

Burnout is a complex construct encompassing clinical, behavioural, and cognitive symptoms. The extensive impact it has on the health and well-being of those affected suggests that burnout may be associated with altered brain activity and structure. For many years, a debate has been underway on whether occupational burnout should be recognised as a disease. This debate does little to improve the quality of life of individuals suffering from burnout. If burnout were formally acknowledged as a disease, governments would allocate greater resources to studying its pathophysiology—facilitating a better understanding of the phenomenon—and would increase spending on diagnosis, treatment, and prevention. It is therefore essential to investigate burnout using various techniques that measure brain parameters. Neuroimaging methods can be beneficial, with magnetic resonance imaging (MRI) being one of the most popular and advanced tools.

Magnetic resonance imaging (MRI) works by aligning abundant tissue ^1^H nuclei in a static field of typically 1.5–3 T, tipping this magnetisation with millisecond radio-frequency pulses, and reading the phase- and amplitude-modulated signal that arises as longitudinal (T_1_) and transverse (T_2_) magnetisation relax while gradient coils impart spatial information; Fourier reconstruction then yields an image in which contrast directly reflects these relaxation properties [19]. The technique is classically divided into structural and functional modes. Structural MRI exploits intrinsic relaxation differences—fat appears hyper-intense on T_1_-weighted scans, water on T_2_/FLAIR—and is further extended by diffusion-weighted and diffusion-tensor sequences that map Brownian motion and white-matter tracts; clinically, diffusion imaging can reveal cytotoxic oedema within minutes of arterial occlusion, guiding ultra-early thrombolysis in acute ischaemic stroke. High-resolution anatomic detail and quantitative morphometry also make structural MRI the reference for tumour delineation and radiotherapy planning, where its superior soft tissue contrast over CT reduces geometric uncertainties and may improve oncological outcomes without ionising radiation [20]. By contrast, functional MRI (fMRI) monitors the blood-oxygen-level-dependent (BOLD) effect: task-evoked neuronal firing triggers a haemodynamic response that transiently lowers deoxyhaemoglobin and hence local susceptibility, producing signal fluctuations that can be modelled into activation maps or resting-state networks; recent work optimising T_2_-prepared BOLD schemes at 3 T and 7 T demonstrates reproducible sub-millimetre resolution, extending fMRI into pre-surgical mapping and network-level phenotyping across neurological and psychiatric disorders [21,22].

Numerous reviews examine burnout’s physiological, neurophysiological, and biological abnormalities [23,24]. However, they focus on multiple health parameters without focusing on neuroimaging findings. Furthermore, these reviews omit most MRI studies on burnout, resulting in a significant gap in understanding the neural correlates of burnout. This mechanistic review aims to identify and synthesize all studies using MRI in burnout. A thorough database search was conducted to identify all relevant studies. However, the aim was not to limit the analysis to a synthesis of results. The most important part of this review is elucidating the potential pathophysiological mechanisms of burnout based on MRI findings. This approach may provide a better understanding of this phenomenon, inform future research, and ultimately contribute to recognizing burnout as a mental disorder.

## 2. Methods

This mechanistic review aims to identify the neural signatures reported in MRI studies of people with clinically diagnosed burnout. We conducted a broad, systematic literature search and applied strict inclusion and exclusion criteria to ensure that the evidence we considered was reliable and relevant. Although our workflow was guided by the core principles of systematic reviews—especially the Preferred Reporting Items for Systematic Reviews and Meta-Analyses (PRISMA)—we tailored several steps for a mechanistic focus, so not every PRISMA component was used exactly as written.

### 2.1. Data Sources and Search Strategy

Reviewers J. Ch. and D. K. separately conducted structured electronic literature searches following predefined standards. Using Boolean logic, they combined the terms “MRI,” “fMRI,” “magnetic resonance imaging,” “magnetic resonance,” “functional magnetic resonance imaging” with “burnout” OR “burnout syndrome.” Searches were run in May 2025 and covered publications dated January 2000 through May 2025. The databases surveyed were PubMed/MEDLINE, ResearchGate, Scopus, Google Scholar, and the Cochrane Library. To ensure comprehensive coverage, the reviewers scanned reference lists of all retrieved papers. They checked PubMed’s “similar articles” suggestions for additional MRI studies on burnout that the primary search might have missed.

### 2.2. Study Selection Criteria

Only clinical trials written in English and published between January 2000 and May 2025 met the eligibility criteria; any papers in other languages were automatically excluded.

### 2.3. Screening Process

We used a multi-stage screening process that kept every study meeting the preset criteria and eliminated those that did not. At each level of review, J. Ch. and K. K. assessed the records independently to preserve impartiality.

#### 2.3.1. Title and Abstract Screening

In the first screening round, both reviewers examined the titles and abstracts of every record in the database searches. At this stage, we confirmed that each study dealt with MRI assessments to meet the inclusion criteria.

#### 2.3.2. Full-Text Assessment

For records that passed the initial title and abstract screening, at this stage, the reviewers verified that each paper was an English-language clinical trial published between January 2000 and May 2025 and that it specifically examined MRI findings in individuals with burnout.

## 3. Results

**Figure 1:**
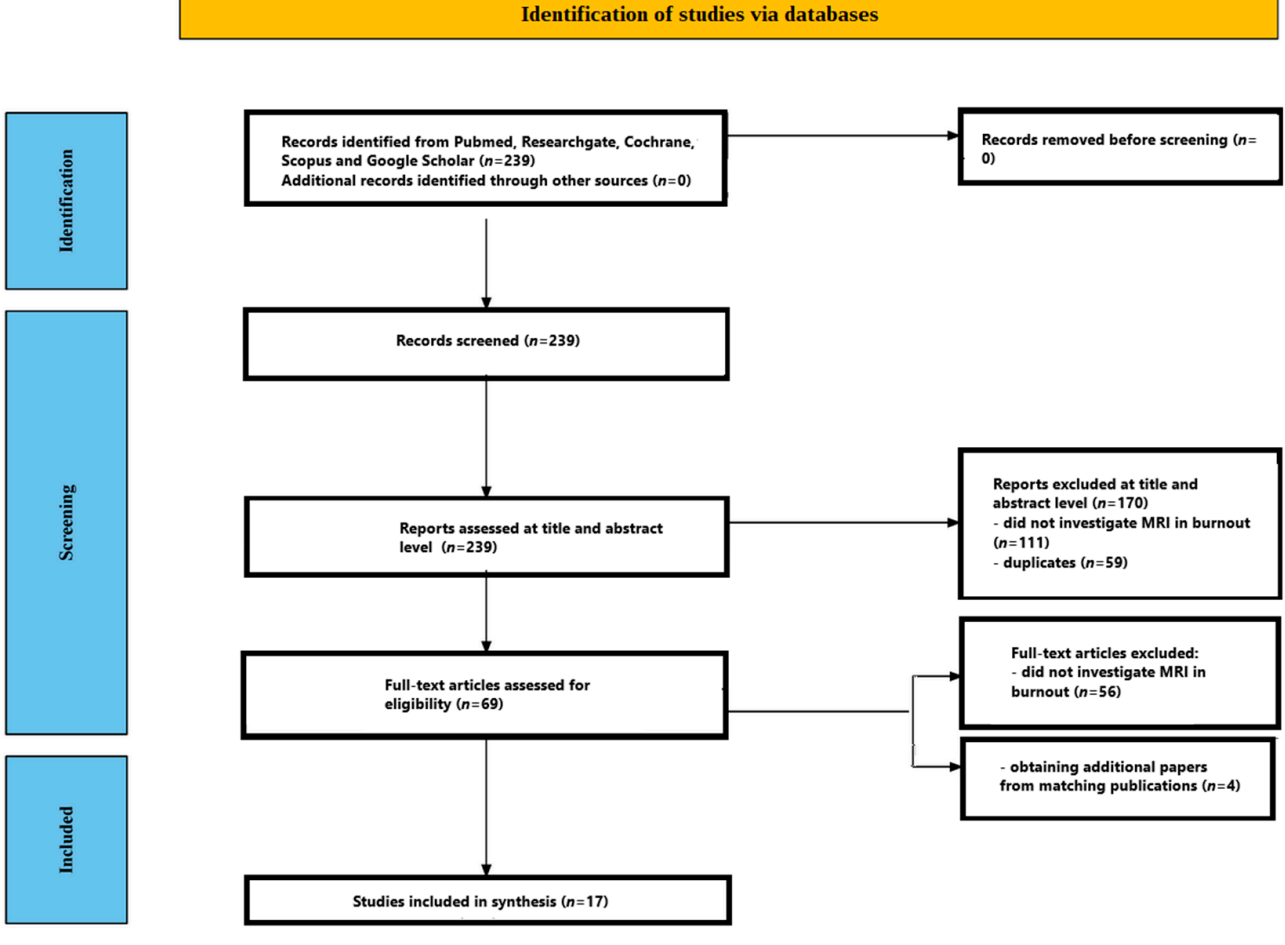
Flow chart depicting the different phases of the systematic review.

Figure 1 illustrates the whole screening process. The initial database search yielded 239 records. After reviewing titles and abstracts, 170 papers were removed—111 did not investigate MRI in burnout, and 59 were duplicates. The remaining 69 articles underwent full-text assessment, and 56 were excluded for not analyzing MRI in the context of burnout. Thirteen studies met all inclusion criteria. A manual search of their reference lists identified four more relevant papers, bringing the final total to 17 studies. All included studies [25,26,27,28,29,30,31,32,33,34,35,36,37,38,39,40,41] are summarized in Table 1.

### 3.1. Participants’ Characteristics

Across the 17 MRI studies that met our inclusion criteria, approximately 1365 individuals were scanned, of whom about 880 exhibited clinically significant burnout or exhaustion and roughly 470 served as healthy or psychiatric comparison participants. Most investigations recruited healthcare professionals: internal-medicine residents and faculty in one study [25], paediatric residents in another [37], and nurses in four reports [27,33,35,41]. The remaining studies examined mixed-occupation employees on sick leave for exhaustion syndrome or adjustment disorder [26,28,29,30,31,34,39,40]. Women constituted nearly 67% of all participants, and nine studies enrolled exclusively or predominantly female samples [27,28,33,34,35,36,38,39,41]. Participants were typically early- to mid-career adults: nurses averaged just over 30 years, mixed-occupation cohorts were generally in their late thirties to early forties, and resident physicians were in their mid-twenties; overall reported age ranges fell between 20 and 46 years.

Methodologically, 14 studies required right-handedness to minimise hemispheric variability [26,27,28,29,30,31,32,33,34,35,36,39,40,41], and every study excluded primary neurological conditions; ten also screened out current DSM-IV/5 psychiatric disorders other than stress-related diagnoses. Burnout was assessed uniformly with the Maslach Burnout Inventory (MBI), though versions varied: eight used the General Survey [26,29,30,31,32,33,36,40], five employed the Human Services Survey or a two-item adaptation [25,27,35,37,38], and the remainder applied validated local exhaustion scales cross-referenced to MBI thresholds [28,34,39]. All studies defined caseness by high emotional exhaustion, high depersonalisation, or reduced personal accomplishment.

Ten investigations included age- and sex-matched healthy controls with low MBI scores [26,29,30,31,32,36,38,39,40,41], while one additionally incorporated psychiatric comparison groups (major depressive disorder) [38]. Longitudinal follow-up was uncommon, appearing only: a 1.5-year post-treatment reassessment of exhaustion-syndrome patients [30] and a 1.5-year surveillance of nurses before and after burnout onset [33]. Collectively, these characteristics indicate that current neuroimaging evidence derives largely from young-to mid-adult, highly educated, predominantly female samples in medical settings; the universal reliance on the MBI facilitates cross-study comparison, yet heterogeneity in cut-off criteria, professional background, and control selection still yields substantial variability in burnout severity across studies.

### 3.2. MRI Acquisition Protocols

The reviewed literature employed diverse magnetic-resonance paradigms that can be grouped into three broad classes—structural morphometry, resting-state connectivity, and task-evoked functional imaging—each probing different facets of burnout-related neurobiology. About 60% of the evidence base is morphometric, 30% interrogates intrinsic connectivity, and only 10–15% employs experimentally controlled fMRI tasks. The heterogeneity of functions-and the limited replication of any single paradigm-suggests that future burnout neuroimaging would benefit from harmonised protocols, multi-centre data sharing, and longitudinal designs capable of separating pre-existing vulnerability from stress-induced change.

#### 3.2.1. Structural Morphometry (8/17 Studies)

Most investigations relied on high-resolution T1-weighted images to quantify grey- and subcortical matter. FreeSurfer-based volumetry or cortical-thickness pipelines dominated [26,28,29,30,31,34,39], targeting stress-sensitive regions such as the amygdala, hippocampus, caudate, putamen, and lateral prefrontal cortex. Two reports used voxel-based morphometry (VBM) in SPM to interrogate whole-brain grey-matter density [27,29]. Field strength was predominantly 3 T, yet three early papers collected data at 1.5 T [29,38,39]; slice thickness ranged from 0.8 to 1 mm, giving sub-millimetre isotropic resolution in the most recent work.

#### 3.2.2. Resting-State Connectivity (4/17 Studies)

Four cohorts examined spontaneous BOLD correlations without an explicit task [33,36,40,41]. Analytical approaches progressed from seed-based (amygdala→cortex coupling in [36]) to whole-brain graph-theoretical metrics [40] and diffusion-map gradient embedding [41]. Two studies extended conventional functional connectivity to effective connectivity using Granger causality [40] or rich-club/feeder–local topology [33]. All were acquired at 3 T with echo times ~30 ms, repetition times 2–2.5 s, and runs of 6–10 min-sufficient to capture low-frequency (<0.1 Hz) fluctuations relevant for network analyses.

#### 3.2.3. Task-Based fMRI (5/17 Studies)

The paradigms varied widely, reflecting heterogeneous clinical questions:

(a) Clinical reasoning: internal-medicine trainees solved USMLE/ABIM multiple-choice cases [25], isolating reading, answering, and post hoc reflection phases.
(b) Psychosocial stress: the ScanSTRESS block design juxtaposed mental arithmetic under social evaluative threat with control blocks [32].
(c) Empathy for pain: nurses viewed video clips of hands receiving painful vs. soft stimuli [35].
(d) Cognitive control: paediatric residents performed an event-related Stroop colour-word task [37].
(e) Working-memory load: women on long-term sick leave completed a 2-back verbal task and a continuous visual-recognition paradigm [38].

Despite their disparity, all tasks converged on fronto-cortical networks—especially the dorsolateral prefrontal cortex, anterior cingulate, and insula—as a common stage on which burnout modulates neural efficiency. Acquisition parameters were homogeneous (3 T, TR ≈ 2 s, whole-brain coverage), but preprocessing packages differed (AFNI in 1, FSL in 13, SPM in 14), complicating direct quantitative synthesis.

#### 3.2.4. Hybrid and Longitudinal Designs

Two studies blended structural and functional approaches within the same cohort: one linked cortical-thickness recovery to improved emotion regulation after 1.5 years of therapy [30]; another paired an emotion-regulation startle task with resting-state connectivity to show that impaired amygdala–ACC coupling tracked behavioural down-regulation deficits [36]. Only two cohorts collected repeat MRI, underscoring the field’s reliance on cross-sectional snapshots.

### 3.3. fMRI Outcomes

Functional findings converge on a small set of cortico-subcortical systems—frontoparietal executive, salience/limbic, default-mode, and sensorimotor hubs—that show burnout-dependent alterations in either the magnitude, efficiency, or temporal dynamics of their BOLD signal.

#### 3.3.1. Task-Evoked Activation

Five experimental fMRI studies implicate the dorsolateral and dorsomedial prefrontal cortices as primary sites of burnout modulation, yet the direction of change varies with task domain and career stage.

During complex clinical reasoning, residents with higher emotional exhaustion recruit the right middle frontal gyrus (BA 6) and right posterior cingulate cortex more strongly when selecting answers, while simultaneously showing reduced precuneus engagement during intuitive processing; none of these effects appear in experienced faculty, suggesting a trainee-specific inefficiency of executive and default-mode switching. Depersonalization was associated with decreased BOLD signal during the answering phase in the bilateral precuneus (BA 7). The decreased BOLD signal was found during the reflecting phase in the right middle frontal gyrus (BA 6) and the right dorsolateral prefrontal cortex (BA 9). When combining both burnout items into a composite burnout score, residents again showed increased activation in the right middle frontal gyrus and right posterior cingulate cortex during the answering phase and decreased activation in the right dorsolateral prefrontal cortex during the reflecting phase. Importantly, no significant associations were found between the faculty group’s burnout scores and brain activity [25].

Under acute psychosocial stress, both controls and burnout participants activate canonical stress circuitry (mPFC, ACC, insula, hippocampus, thalamus, striatum), but only controls down-regulate dorsal ACC activity over repeated blocks; individuals with burnout instead show a progressive increase in dACC signal, betraying a failure of adaptive dampening [32].

In attention-demanding conflict burnout, participants showed that congruent and incongruent conditions activated canonical attention-related regions, including bilateral dorsolateral prefrontal cortex (DLPFC), anterior cingulate cortex (ACC), and inferior parietal lobules. However, the incongruent condition elicited more extensive activation, as expected due to its greater attentional demand. Crucially, the contrast between incongruent and congruent conditions (Stroop effect) showed a positive correlation between burnout scores (emotional exhaustion and depersonalization combined) and increased BOLD response in the right middle frontal gyrus, a core region of the DLPFC. This correlation remained significant after controlling for VAMS anxiety scores, confirming that the observed effect was specific to burnout and not simply a byproduct of mood or transient anxiety [37].

The first captured task in [38] engagement across all groups, with occipital and prefrontal regions activated more during memory tasks than baseline. However, only the control and depression groups showed significant frontal activation during the 2-back task. In contrast, the LTSL group’s prefrontal response to the 2-back task was absent. The second latent variable distinguished the two memory tasks. The 2-back task was associated with a frontoparietal network, while the CVMT activated dorsal occipital and temporal regions. This pattern was consistent across all groups. Univariate analysis confirmed no significant group differences during the visual memory task. However, during the 2-back task, LTSL participants showed significantly reduced activation in the left ventrolateral and right dorsolateral prefrontal cortex.

The study [35] analysis revealed that in response to painful stimuli, significant brain activation occurred in the AI/IFG and TPJ regions. However, the ACC did not show sufficient activity to meet the statistical threshold and was excluded from further analysis. The researchers found a consistent and significant negative correlation between empathy-related brain activity (in AI/IFG and TPJ) and burnout severity. In other words, the more exhausted the participants reported feeling, the less activation was observed in these empathy-related brain regions. Similar negative correlations were found between brain activity and scores for emotional dissonance, alexithymia, and dispositional empathy.

#### 3.3.2. Rest-State Connectivity

Four independent cohorts show chronic occupational stress degrades the brain’s intrinsic communication architecture.

The burnout group in [36] showed weaker connectivity from the amygdala to the mPFC, dlPFC, and motor cortex. Conversely, they showed stronger connectivity between the amygdala and insula (bilaterally) and the cerebellum (especially the vermis and anterior regions). Further analysis revealed that stronger connectivity between the amygdala and ACC was associated with a greater ability to down-regulate negative emotion. Stress severity (MBI-GS scores) correlated positively with connectivity between the left amygdala and the insula and hypothalamic-thalamic region.

Longitudinal rich-club mapping in nurses shows global loss of connection strength-rich-hub, feeder, and local alike-after burnout emerges, with mid- and long-range edges most affected and a vulnerable sub-network centred on precuneus and basal ganglia that tracks worsening emotional exhaustion [33].

Both the burnout and control groups exhibited small-world organization for global topology, a balance between local specialization and global integration commonly seen in healthy brain networks. However, participants with burnout showed significantly increased characteristic path length (Lp) and normalized path length (k), along with decreased global efficiency (Eglob), suggesting a disruption in global network integration and a shift toward a more regular (less efficient) brain network structure. At the nodal level, significant differences emerged in regions primarily within the visual network and in the auditory and limbic networks. The burnout group demonstrated increased degree centrality and nodal efficiency in the bilateral cuneus and left superior occipital gyrus, indicating heightened local connectivity in visual areas. Increased betweenness centrality was observed in the right superior temporal gyrus, an auditory region involved in language and stress processing.

Meanwhile, decreased centrality and efficiency were found in the left anterior cingulate cortex (ACC), a limbic structure implicated in attention, emotion regulation, and conflict monitoring. Notably, the left fusiform gyrus (FFG.L), associated with visual recognition, showed increased local efficiency in the burnout group. Using the directed Network-Based Statistic (dNBS) method, the study identified a subnetwork with significantly decreased effective connectivity in the burnout group, involving 12 brain regions and 14 directed edges. Most of these disrupted connections originated from the visual network and terminated in the right hippocampus, a structure critical for memory and emotional regulation. Other regions in this dysfunctional subnetwork included the right supramarginal gyrus, medial orbitofrontal cortex, and middle temporal gyrus, indicating broader disruptions across memory, default mode, and cognitive control networks. Correlation analyses further revealed that depression severity (measured by SDS) was negatively associated with both characteristic path length and the nodal local efficiency of the fusiform gyrus. However, these correlations remained insignificant after correction for multiple comparisons [40].

Manifold-learning gradients further reveal left-hemispheric expansion of functional eccentricity in somatomotor and visual networks; the spatial pattern aligns with cortical expression of genes governing circadian rhythm (positive association) and astrocytic synaptic maintenance (negative), linking connectome distortion to molecular stress pathways [41].

### 3.4. Structural MRI Outcomes

The morphometric literature describes a relatively consistent pattern of stress-related change centred on three neuroanatomical axes—(i) limbic hypertrophy of the amygdala, (ii) striatal and prefrontal atrophy, and (iii) selective sex- and time-dependent reversibility—while in striking contrast to trauma research, the hippocampus is largely spared.

#### 3.4.1. Amygdala: Hypertrophy, Female-Specific

Across five independent cohorts, the amygdala is the single most replicated site of structural enlargement. In two Swedish Exhaustion-Syndrome datasets, women showed bilateral expansion of the basolateral and central nuclei, and positive correlations with perceived stress [26,30]. A voxel-based morphometry study of mixed-sex employees likewise reported right-predominant amygdala hypertrophy [31]. Crucially, none of these studies detected amygdala change in men, and one documented a significant diagnosis × sex interaction [30], supporting a hormone-modulated vulnerability model.

#### 3.4.2. Striatum: Caudate and Putamen Atrophy, Male-Biased

Reductions in dorsal striatal volume are almost as robust as amygdala enlargement but show the opposite sex gradient. Manual volumetry and VBM revealed bilaterally smaller caudate and putamen in chronically stressed employees [29,31], while in the considerable Exhaustion-Syndrome cohort, caudate shrinkage reached significance only in men [30]. High mental fatigue in rehabilitation patients was likewise linked to diminished caudate and putamen volumes after controlling for depression [28]. These convergent findings implicate fronto-striatal circuitry—central to effort–reward calibration—as a structural target of occupational stress, particularly in males.

#### 3.4.3. Prefrontal Cortex: Thinning or Reduced Grey-Matter Density

Three studies reported focal loss of cortical thickness or grey-matter volume in prefrontal regions that orchestrate executive control and emotion regulation. Nurses with high emotional exhaustion exhibited reduced grey matter in bilateral ventromedial PFC and left insula, with additional middle-frontal loss at liberal thresholds. Similarly, higher depersonalization scores were negatively correlated with GM volume in the left vmPFC [27]. Employees with adjustment disorder showed widespread thinning of the medial PFC proportional to age and stress load [31]. In Exhaustion-Syndrome, cortical thinning localised to left superior frontal, superior temporal, and opercular cortices-again most pronounced in women [30]. An extensive dimensional study (n = 300) found a weak positive association between perceived stress and left lateral PFC thickness [34], hinting that early or compensatory hypertrophy might precede overt atrophy in cross-sectional snapshots.

#### 3.4.4. Hippocampus: Largely Intact

Across the burnout-MRI corpus, the hippocampus emerges as a striking neuro-anatomical exception: every study that measured it, whether through whole-structure voxel-based morphometry, automated FreeSurfer sub-field segmentation, or painstaking manual tracing, reported null effects.

The volumetric work in mixed-sex exhaustion-syndrome samples [26] found that neither total hippocampal volume nor any of its CA1–CA4/dentate sub-fields differed from matched controls, in women or men. Subsequent VBM in nurses [27] likewise showed no association between grey-matter density in the hippocampus and individual variation in emotional exhaustion or depersonalisation, even though parallel analyses detected robust atrophy in the ventromedial prefrontal cortex and insula. Manual volumetry in chronically over-worked office professionals [29] corroborated the picture: caudate and putamen were significantly smaller, but hippocampal size was indistinguishable from that of controls. The only longitudinal cohort [30] scanned patients at diagnosis and 1.5 years into rehabilitation. Volumes of the caudate and prefrontal cortex normalised as symptoms abated, yet the hippocampus remained unchanged at both time-points, confirming structural stability over the course of recovery. A separate FreeSurfer study of long-term occupational stress [31] again observed amygdala hypertrophy and caudate reduction without hippocampal involvement. Finally, high-resolution sub-field measurements in women with work-related exhaustion [39] showed intact CA1 and CA2/3 volumes despite pronounced attentional and memory deficits.

Taken together, these convergent null findings—spanning scanner strengths, segmentation methods, clinical populations, and cross-sectional as well as longitudinal designs—indicate that chronic occupational burnout does not produce the hippocampal atrophy so characteristic of post-traumatic stress disorder or major depression. Burnout’s structural signature instead centres on prefrontal, striatal, and (in women) amygdalar circuits, suggesting a pathophysiology that spares medial-temporal integrity. Whether subtler micro-architectural or connectivity changes exist remains an open question for ultra-high-field imaging and diffusion techniques. Still, current morphometric evidence portrays the hippocampus as structurally resilient in prolonged work-related stress.

#### 3.4.5. Other Cortical and Cerebellar Findings

Isolated reports describe thicker occipital cortex in female patients [30], reduced anterior cingulate grey matter density [29], and heightened local efficiency in fusiform and superior occipital areas on graph-theoretical metrics [40].

### 3.5. Correlation of MRI Findings with Cognitive and Behavioural Outcomes

Across the papers that reported explicit brain–behaviour links, each was supported by specific study numbers.

#### 3.5.1. Compensatory Executive Overdrive

Early-stage burnout is typically managed by pushing the executive network harder. Internal-medicine residents with higher emotional-exhaustion scores showed larger BOLD responses in the right middle-frontal gyrus and posterior cingulate while answering diagnostic questions, yet achieved no accuracy gain [25]. Paediatric residents displayed a linear rise in right-DLPFC activation during Stroop conflict as the joint emotional-exhaustion + depersonalisation score increased; the strongest signal was coupled with the slowest responses, revealing neural inefficiency [37]. By contrast, women on long-term sick leave for exhaustion recruited left ventro-/dorsolateral PFC less during a 2-back task, and lower activation predicted slower reactions, indicating under-mobilisation once burnout becomes chronic [38].

#### 3.5.2. Limbic Dysregulation of Emotion and Empathy

Weakened amygdala–ACC/mPFC coupling predicted larger startle responses and poorer self-rated success when down-regulating negative emotion [36]. In the ScanSTRESS paradigm, burnout severity did not alter mean response but abolished the normal decline of dorsal-ACC activity over repeated stress blocks-exhausted participants showed a paradoxical escalation [8]. Empathy data showed the “emotional-dissonance” paradox: nurses with higher exhaustion and depersonalisation but self-declared high empathy exhibited blunted anterior-insula/IFG and TPJ responses to others’ pain; the greater the self–other distress gap, the lower the neural response [35].

#### 3.5.3. Striato-Frontal Control of Mental Fatigue, Memory, and Reward

Smaller caudate and putamen volumes correlated negatively with subjective fatigue in high-fatigue ED patients [28] and mixed-occupation stress samples [29,31]. In study [28], mental-fatigue scores mediated the link between caudate size and working-memory accuracy. Longitudinal data showed that caudate volume recovered after multimodal therapy, and this recovery paralleled normalisation of emotion-regulation reflexes, whereas persistent amygdala enlargement accompanied lingering anxiety [30]. Sex specificity was apparent: striatal shrinkage was strongest in men [30], whereas amygdala hypertrophy tracked stress only in women [26,30].

#### 3.5.4. Network Integrity as a Barometer of Clinical Course

In nurses followed for 18 months, the steepest losses in mid- and long-range functional connectivity centred on the precuneus and basal ganglia correlated almost one-to-one with rises in emotional-exhaustion and anxiety [33]. Whole-brain graph metrics linked higher depression scores with longer characteristic path length and lower global efficiency [40]. Manifold-learning gradients showed that expansion of functional “eccentricity” in left somatomotor cortex scaled with depersonalisation and emotional exhaustion [41].

### 3.6. Neuro-Endocrine and Molecular Correlates

A complementary thread running through the imaging literature links the observed brain alterations to stress-hormone signalling, immune activation, and transcriptomic signatures, suggesting that burnout’s neural footprint is embedded in a broader biological cascade.

#### 3.6.1. HPA-Axis Markers

Five studies collected endocrine data in parallel with MRI. The ScanSTRESS paradigm triggered typical salivary-cortisol rises in a large non-clinical burnout sample. Yet, neither the peak nor the recovery slope differed from controls [32], implying that neural dysregulation can precede frank HPA-axis change. By contrast, women on long-term sick leave showed a flattened diurnal cortisol curve, especially a blunted early-morning drop, without gross hyper- or hypo-cortisolaemia [38]. More fine-grained pharmacological probes reveal subtler dysfunction: patients with Exhaustion Syndrome exhibited a blunted ACTH surge to CRH but normal dexamethasone suppression and Synacthen responses [39], pointing to reduced pituitary drive rather than adrenal failure.

#### 3.6.2. Immune and Inflammatory Signals

Elevated IL-1β and low-grade systemic inflammation characterised the same Exhaustion-Syndrome cohort [39]. They coincided with smaller prefrontal volumes and poorer visuospatial memory, dovetailing with pre-clinical data in which pro-inflammatory cytokines disrupt synaptic plasticity. Although imaging-wide neuro-immune mapping remains sparse, these findings suggest chronic work stress may engage a cytokine-mediated route to grey-matter loss.

#### 3.6.3. Transcriptomic Alignment

The gradient-mapping investigation in nurses [41] spatially matched functional-connectome distortions to cortical gene-expression maps from the Allen Human Brain Atlas. Two gene sets emerged: a PLS^+^ cluster enriched for circadian-rhythm genes whose high expression overlapped somatomotor areas showing eccentricity expansion, and a PLS^−^ cluster enriched for astrocytic synaptic-maintenance genes whose low expression colocalised with connectivity loss. These patterns imply that shift-work-driven circadian disruption and impaired glial support may jointly sculpt burnout-related network re-organisation.

## 4. Discussion

Burnout is a condition characterized by decreased productivity, depressed mood, increased stress, cynicism, and many other unpleasant symptoms. This complex kaleidoscope of symptoms rightly raises the suspicion that individuals with burnout have altered brain function, and perhaps even structure. Medicine and neuroscience utilize advanced tools such as magnetic resonance imaging (MRI) to study the brain in various contexts. This study aimed to identify and analyze all MRI studies to assess individuals with burnout.

A general review of the studies reveals several facts. First, 17 studies demonstrate that burnout is attracting the scientific community’s attention, which is not afraid of bearing the economic costs of studying a condition not recognized as a disease in disease classifications. The number of studies allowed for collecting a total group of over 1000 patients with burnout, a significant number that allows for the formulation of reliable conclusions and observations. Second, the studies do not focus solely on neuroimaging measurements but utilize neurochemical, cardiac, and other measures. This approach allows for obtaining more comprehensive health data on participants and correlating them with brain scan results. Third, and most importantly, the brain of a person with burnout differs significantly from that of a healthy person, both functionally and structurally. These differences are discussed in greater detail in the following sections, and mechanistic explanations for the pathophysiology of burnout are provided. Evidence from neuroimaging studies, a robust form of collecting data on brain function, provides evidence that burnout is characterized by cortical and subcortical brain dysfunction. It should finally be recognized as a disease and adequately funded for its research, diagnosis, treatment, and prevention. Below, we discuss the findings from MRI studies, attempting to elucidate the pathophysiological mechanisms and place the results in a broader clinical context.

### 4.1. Amygdala Volume, Stress, HPA Imbalance

Studies [26,30,31] showed increased amygdala volume, which may depend on gender; in women, the amygdala volume was larger. In [31], the stressed group showed a bilateral amygdala enlargement, with the right side particularly affected. The amygdala, known for its role in processing fear and emotional salience, was positively correlated with perceived stress levels, meaning the higher the stress score, the larger the amygdala. The amygdala sits at the centre of the brain’s stress network. Sensory, contextual and pre-frontal inputs converge on the basolateral amygdala (BLA), whose excitatory output drives the central and medial nuclei and, through them, the hypothalamic–pituitary–adrenal (HPA) axis and sympathetic responses that characterise the physiological stress state [42]. Functional imaging confirms that this circuitry becomes over-reactive in stress-related disorders such as PTSD, anxiety, and depression, and the degree of hyper-activation parallels symptom severity [42]. Pre-frontal regions usually dampen the amygdala, but chronic stress weakens that top-down control, and females in particular show stronger amygdala-PFC engagement when processing negative cues, highlighting a vital sex difference in the stress network [43].

Long-lasting stress reshapes the amygdala at every biological level. In animal models, repeated restraint, immobilisation, or social defeat triggers exuberant dendritic growth and persistent spine addition in BLA principal neurons, whereas hippocampal and medial-prefrontal neurons simultaneously retract [44]. These structural changes are accompanied by a shift in synaptic balance: glutamatergic drive onto BLA cells increases. At the same time, GABAergic tone falls, creating a chronic excitation–inhibition imbalance that leaves the network hypersensitive to subsequent threats. Sustained glucocorticoid exposure is a key trigger. Activating glucocorticoid receptors in the amygdala upregulates corticotropin-releasing factor, disrupts GABA signalling, and further potentiates HPA output, establishing a feed-forward loop between the amygdala and endocrine stress system [45]. Many studies have shown that people with burnout have an HPA imbalance and elevated cortisol levels [46,47,48,49,50,51,52,53,54,55,56,57,58]. Parallel neuro-immune changes-microglial activation and pro-inflammatory cytokines-reinforce excitatory synaptic scaling and may contribute additional volume to the tissue [59]. Neuroinflammation has also been demonstrated in burnout [60,61,62].

These mechanisms explain why magnetic-resonance studies of occupational burnout—essentially a model of chronic, uncontrollable psychosocial stress—reveal larger total amygdala volumes that correlate with higher exhaustion and perceived-stress scores. Dendritic hypertrophy, ongoing synaptogenesis, and glial swelling collectively enlarge grey-matter volume [63], while persistent hyper-excitability locks the network into an energy-demanding state that sustains negative affect and vigilance [59]. Sex hormones modulate this plasticity: oestrogen and progesterone both amplify brain-derived neurotrophic factor–dependent spine formation [64,65,66], and women exhibit more vigorous stress-evoked amygdala activity than men [67], providing a plausible substrate for the more pronounced volumetric increase reported in female burnout sufferers. An enlarged, hyper-responsive amygdala has several clinical consequences. Sending stronger excitatory projections to the paraventricular hypothals prolongs cortisol release, delaying physiological recovery from daily hassles. Within cognitive–emotional circuits, the same hyperactivity biases attention towards threat, impairs executive control, and accelerates the spiral of fatigue, cynicism, and inefficacy that defines burnout. The sex-specific structural differences identified in the amygdala have direct behavioral and clinical correlates. This pattern may help explain epidemiological data showing higher burnout prevalence and stress-related affective symptoms in female healthcare professionals [68,69,70].

### 4.2. Roles of Frontal Gyrus and Precuneus

In study [25] among resident physicians—but not seasoned faculty—higher emotional-exhaustion scores predicted greater BOLD signal in the right middle frontal gyrus (MFG; BA 6) and posterior cingulate cortex (PCC; BA 31) while solving diagnostic problems, whereas higher depersonalization scores predicted lower activity in the bilateral precuneus (BA 7) during problem solving and reduced right dorsolateral prefrontal cortex (DLPFC; BA 9) engagement during subsequent reflection. A composite burnout index reproduced this “hyper-MFG/PCC, hypo-DLPFC/precuneus” profile in trainees, but no parallel associations emerged in faculty.

BA 6 intersects premotor planning [71] and the executive control network [72]. During progressively harder n-back and mental-arithmetic tasks, healthy volunteers show a monotonic rise in proper MFG BOLD that mirrors working-memory load [73]. Acute psychosocial stress further strengthens PCC connectivity with limbic salience hubs and elevates absolute PCC activity, suggesting that default-mode nuclei are pulled “online” when allostatic demand rises [74]. The resident data likely reflect a costly strategy in which extra premotor/executive resources are recruited but must contend with an incompletely suppressed PCC, consistent with cognitive-load theory’s claim that extraneous affective load consumes limited control capacity.

The precuneus (BA 7) is central to schema retrieval [75], visuospatial imagery [76], and non-analytic pattern completion [77]; hypoactivity in this region accompanies dissociative derealization states [78]. Simultaneous under-recruitment of the right DLPFC mirrors trait-level deficits seen in bipolar disorder, schizophrenia, and other conditions marked by motivational disengagement, where reduced BA 9 activity co-exists with intact behavioral accuracy yet heralds poorer metacognitive monitoring [79,80]. The burnout signature, therefore, resembles a dual hit: intuitive pattern-matching (precuneus) and deliberative working-memory (DLPFC) circuits are both throttled, leaving residents to rely on slower, effortful control processes.

Faculty may engage neural networks more efficiently due to more developed illness scripts and greater automation of diagnostic processes. The authors relate these findings to cognitive load theory, which posits that working memory has limited capacity. Burnout-particularly emotional exhaustion-may impose extraneous cognitive load, consuming mental resources needed for clinical tasks. This is evidenced by increased but inefficient neural activation in specific brain regions among residents. In contrast, depersonalization may suppress brain activity in areas vital for intuitive and deliberate reasoning, suggesting a dual impairment. These findings are interpreted as evidence that burnout interferes with the neurological basis of clinical reasoning in physicians, particularly trainees. The study supports the view that residents are more neurologically vulnerable to burnout and would benefit from emotional support and interventions to reduce cognitive strain.

### 4.3. Volumes of vmPFC, Insula, and Thalamus

The study [27] showed a decrease in the ventromedial prefrontal cortex (vmPFC) volume. Chronic occupational burnout can be thought of as a slow-motion stress experiment run on the human brain daily. The first domino to fall is neuroendocrine: repeated feelings of demand outstripping control keep the hypothalamic-pituitary-adrenal axis humming, bathing the cortex in glucocorticoids and catecholamines. In pyramidal neurons of the vmPFC and dorsolateral middle frontal gyrus, high corticosterone and noradrenergic drive open calcium- and cAMP-gated potassium channels that silence synapses; with chronic exposure, the same signalling cascade prunes dendritic spines, shrinks apical arbors and ultimately erodes regional gray-matter volume-changes documented in both rodent stress models and human neuroimaging [81,82]. The voxel-based morphometry study shows exactly this footprint: higher emotional-exhaustion scores track linearly with smaller vmPFC volumes, and an exploratory threshold reveals parallel loss in the left middle frontal gyrus, confirming that structural attrition progresses along the prefrontal control hierarchy.

Why does that matter clinically? The vmPFC is the main cortical brake on threat circuitry; it sends inhibitory projections to the amygdala, hypothalamus, and brainstem that quell autonomic arousal and negative affect [83,84,85]. When synaptic density in the vmPFC is whittled away, the brake pads thin: cortisol peaks last longer, heart-rate variability falls [86,87,88], and the subjective sense of being unable to “wind down” blossoms into emotional exhaustion and, later, cynicism. Functional work corroborates the structural picture: resting-state EEG in burned-out employees shows weakened frontal and midline connectivity in the alpha band [89,90], a possible neural signature of diminished top-down governance over limbic and default-mode hubs.

A second, complementary pathway runs through the anterior insula. This cortex integrates interoceptive signals with social-emotional context, generating the visceral “feel” of another person’s state [91,92,93]. The study [27] results link higher exhaustion scores to reduced left-insula volume. Mechanistically, the insula is rich in interleukin receptors and microglia [94,95]; chronic allostatic load upregulates inflammatory cascades here, accelerating synaptic stripping as much as it does in systemic inflammatory pain disorders [96]. When the insula’s granular layers lose cells and connections, clinicians report exactly what the MRI predicts: dulled interoception (skipped meals and unnoticed tachycardia) and a colder empathic stance toward patients.

The third node, the thalamus, acts as the brain’s sensory relay and arousal modulator. Elevated depersonalization on the Maslach inventory correlates with smaller left thalamic volume in the dataset, echoing rodent work in which a single-prolonged stress paradigm produces bilateral thalamic atrophy accompanied by microglial activation [97].

The vmPFC, insula, and thalamus are densely interconnected hubs within cortico–subcortical control networks, linked by long-range white-matter pathways that integrate salience detection, interoceptive awareness, and executive regulation [98,99]. The thalamus is a master regulator of cortical communication, modulating activity within and between large-scale networks [100]. In contrast, the insula relays viscerosensory input from thalamic nuclei to prefrontal regions, enabling adaptive switching between internal and external task demands [99]. Disruption of this “regulatory triangle” will likely impair global network efficiency and top-down control. In burnout, such disconnection is mirrored by reductions in frontal and midline alpha-band coherence on EEG—a rhythm strongly implicated in attentional gating and working-memory maintenance [90,101]. This loss of synchronized alpha activity is associated with slower decision-making, reduced cognitive flexibility, and the working-memory lapses and decision fatigue that can precipitate prolonged occupational disengagement and sick leave.

### 4.4. Dysfunction of the Reward System

The markedly smaller caudate and putamen volumes in the high-fatigue group in the study [28] replicate a pattern already described in several independent burnout and exhaustion-disorder cohorts, where chronic occupational stress selectively erodes dorsal-striatal grey matter while leaving many cortical indices intact. The striatum—especially its dorsal (caudate/putamen) sector—acts as the hub of three fronto-striatal loops (motor, cognitive, limbic) [102,103]. Volumetric loss, therefore, signals a reduced structural “bandwidth” for transmitting cognitive-control commands and reward-prediction signals from cortex to thalamus and back. Moreover, in [31], the reduction of caudate nucleus volume correlated negatively with exhaustion scores. It was accompanied by impaired performance in the pegboard test, especially with the dominant hand, indicating functional consequences of the structural changes.

Striatal dopamine normally biases behaviour toward actions whose benefits outweigh their subjective costs. Converging PET, fMRI, and pharmacological data show that dampening dorsal-striatal dopamine makes effort feel more expensive and induces motivational fatigue, whereas boosting dopamine has the opposite effect [104,105]. Because sustained stress downregulates D_2_-receptor signalling and shrinks medium spiny neurons [106,107,108], a smaller caudate most likely indexes a chronic dopaminergic shortfall that inflates the perceived cost of mental work, even when objective performance remains possible. The negative caudate-volume → mental-fatigue correlation, coupled with the mediation result, can be formalised as a cost-control loop: “smaller caudate → less dopaminergic gain on benefits → inflated cost signal → higher subjective fatigue”. Participants then choose one of two strategies: decrease output (seen in Parkinson’s and MS) or retain output by over-recruiting executive resources. Burnout patients take the latter path, consistent with real-life reports of “pushing through” exhaustion.

Inverted U models of dopamine predict a performance optimum just below receptor saturation; converging animal and human evidence shows that working-memory accuracy is maximal at intermediate D1/D2 stimulation and declines when dopamine is either depleted or excessive [109,110,111]. If chronic stress pushes an individual onto the left, hypodopaminergic limb of that curve-something repeatedly documented because of prolonged stress exposure-baseline dopamine tone falls below the optimum [112,113]. Under those conditions, the burst-like (phasic) dopamine released when the person must “push” themselves to perform can nudge the system back toward the peak of the curve, temporarily improving working-memory performance even while the subjective cost of effort (fatigue) mounts [114]. The mediation analysis captures this indirect rescue effect. In other words, fatigue is the conscious cost of the extra dopaminergic “push” that keeps performance afloat.

The data map neatly onto the original basal-ganglia model of central fatigue, which posits that impaired integration of limbic (motivation), cognitive (plan selection), and motor (execution) inputs within the striatum undermines the energisation of goal-directed behaviour [115]. The present findings refine that framework by showing that subjective fatigue can rise long before overt cognitive failure, because individuals may “buy” performance with costly compensatory effort when cortical resources remain available.

### 4.5. Left STG Thinning

On MRI, chronic-occupational burnout has been linked to reduced cortical thickness in the left superior temporal gyrus (STG)-a region whose standard function anchors several high-demand cognitive operations [30]. The posterior left STG, bordering Wernicke’s area, provides the primary cortical gateway from acoustic-phonetic features to lexical–semantic representations [116], activates whenever listeners decode meaningful speech in multitalker settings [117], and underpins auditory short-term memory capacity and sentence comprehension; lesions here cause classic receptive aphasia [118]. More dorsal STG fields (including Heschl’s gyrus) parse pitch, rhythm, and timbre and help segregate attended speech from background noise. At the same time, the upper bank of the superior temporal sulcus contributes to mental-state attribution and the reading of emotional prosody, tightly coupling the STG to the amygdala–prefrontal “social brain” network. These convergent observations place the left STG at the crossroads of language, auditory scene analysis, and socio-emotional decoding-domains taxed to saturation in many burnout-producing jobs [118,119,120].

Cortical thickness is the MRI-distance between pial and white-matter boundaries; its reduction can arise from several microstructural processes. Pro-inflammatory cytokines and glucocorticoids shrink dendritic arbors and prune synapses, reducing neuropil volume. Conversely, progressive intracortical myelination, especially during late development, can make gray matter look thinner by inward shifting the gray–white border. Age- and disease-related neuronal loss, glial remodeling, and changes in extracellular matrix all contribute additional variance, so “thinning” is best viewed as a composite indicator of altered cyto- and myeloarchitecture rather than direct neuron counts [121,122,123].

Burnout offers a plausible pathophysiological route to left-STG thinning. Prolonged hypothalamic–pituitary–adrenal activation keeps cortisol tonically elevated; the STG expresses dense glucocorticoid receptors, making it vulnerable to stress-induced synaptic retraction and glial change. At the same time, many service and educational occupations impose continuous auditory vigilance and emotionally laden conversation, driving sustained metabolic demand in exactly the language–auditory circuitry centred on the left STG. Imaging studies of exhaustion syndrome confirm this convergence: patients show left-STG thinning alongside medial-prefrontal thinning and amygdala enlargement, and these abnormalities scale with perceived stress and partially recover only after months of treatment, suggesting that early changes may be adaptive but become entrenched when overload persists. Independent literature links stressful life events and cortisol reactivity to accelerated thinning in temporal cortices, reinforcing a stress-neurotoxicity model [124,125,126].

### 4.6. Abnormal dACC Response to Stress

In the study [32], a novel and significant finding emerged when researchers examined the exposure-time effect. Specifically, while the HC group showed decreasing activation over time in the left dorsal anterior cingulate cortex (dACC)-interpreted as a healthy, adaptive dampening of neural stress response-the burnout group displayed the opposite pattern: increasing activation over time in the same region. This group-by-time interaction was statistically significant and suggests that individuals with burnout may have a reduced ability to downregulate brain responses during prolonged or repeated stress exposure. This altered exposure-time pattern in the dACC is particularly noteworthy because this brain region plays a key role in cognitive control [127], emotional regulation [128], and stress appraisal [128]. The finding aligns with previous research in patients with major depressive disorder [129]. It supports the idea that temporal dynamics of neural activation (how it changes over time), rather than static snapshots of activation magnitude, may be critical markers of stress-related dysfunction. The researchers suggest that this pattern-a lack of “neuroflexibility”-might be a subtle early neural signature of burnout before it reaches clinical severity. Despite the robustness of the exposure-time effect finding, the study did not observe broader group differences in brain regions like the amygdala, which had been implicated in prior burnout-related research. Potential explanations for the limited findings include the moderate severity of burnout in the BO group, sample heterogeneity in age and hormonal status, and the block-design nature of the ScanSTRESS task, which may dampen cortisol responses compared to single-shot stress tasks like the Trier Social Stress Test.

### 4.7. Rich-Club Weakening Captures a Systems-Level Cost of Burnout

Study [33] demonstrates that nurses transitioning from health to burnout over 18 months lose strength across rich-club, feeder, and local edges, with mid- and long-range functional links showing the steepest decline. Rich-club connections are metabolically expensive “highways” that integrate distributed modules; they are preferentially preserved in normal development and ageing, yet become vulnerable under energetic strain [130,131]. Acute psychosocial stress already suppresses whole-brain efficiency, implying a rapid, cortisol-driven throttling of high-cost edges [132]. Chronic workplace stress likely extends this transient downregulation, eroding synaptic strength along myelinated long-range tracts, as observed in small-vessel disease and mid-life hypertension, where rich-club degradation tracks cognitive decline [133]. The rich-club findings of the study [33] therefore fit a broader “metabolic cost–capacity” model in which sustained glucocorticoid exposure, neuroinflammation, and impaired neurovascular coupling converge on the brain’s most energy-hungry links.

The precuneus lies at the intersection of the default-mode, attentional, and memory systems; its disconnection aligns with depersonalisation and autobiographical memory complaints typical of burnout. Basal-ganglia edges—particularly putamen—parietal links noted in study [33]-are essential for reward prediction and effort allocation; their weakening echoes motivation loss and fatigue, a pattern mirrored in multiple-sclerosis fatigue, where striatal connectivity predicts subjective tiredness [134]. Together, these data suggest that burnout is less a focal “limbic” problem than a distributed networkopathy in which cortico-striato-parietal loops fail to sustain self-referential and motivational processing.

Aberrant default-mode dominance has emerged as a transdiagnostic stress marker. Resilience correlates with preserved medial prefrontal–posterior cingulate connectivity [135]. Fire-fighter cohorts show parallel shifts toward salience-network hyper-coupling and default-mode hypoconnectivity under occupational stress [136]. The longitudinal collapse of rich-club efficiency in study [33] may thus tip an already labile default-mode/salience balance, amplifying interoceptive threat monitoring at the expense of reflective distance-an interpretation consistent with the strong anxiety-connectivity correlations reported [137].

### 4.8. Burnout and Empathy

In the experiment [35] the authors aimed to test two competing theories about the causes of burnout: the compassion fatigue hypothesis, which suggests that burnout arises from excessive emotional involvement and high empathy, and the emotional dissonance hypothesis, which proposes that burnout is caused by a conflict between what individuals feel and what they are expected to express, often linked to reduced emotional regulation or awareness (alexithymia). The researchers found a consistent and significant negative correlation between empathy-related brain activity (in AI/IFG and TPJ) and burnout severity. In other words, the more exhausted the participants reported feeling, the less activation was observed in these empathy-related brain regions. Similar negative correlations were found between brain activity and scores for emotional dissonance, alexithymia, and dispositional empathy. These findings suggested a complex pattern: individuals who reported higher empathy, greater difficulty identifying their emotions, and greater emotional dissonance also exhibited lower neural responses to others’ pain. This undermines the compassion fatigue theory, which predicts burnout from excessive empathy, and instead supports the emotional dissonance theory [138]. The key point is that while participants may report being highly empathic, their brains may show reduced activation in empathy-related areas due to emotional suppression or lack of emotional clarity, typical features of alexithymia. Further analysis of post-scan ratings, where participants compared how distressed they felt watching the clips versus how painful they believed the videos were for others, also revealed that a greater discrepancy (i.e., emotional dissonance) correlated with lower brain activation and higher burnout. This reinforced the idea that burnout is not a simple outcome of too much empathy, but rather a result of impaired emotional processing or regulation. The researchers discussed the role of the IFG in modulating negative emotions and promoting cognitive control, as well as the AI’s function in emotional awareness. Reduced activity in these areas may impair the ability to manage emotional responses effectively. Similarly, the TPJ is implicated in distinguishing self from others [139], perspective-taking [140,141], and theory of mind [142,143]. Diminished TPJ activity might weaken this boundary, increase emotional confusion, and lead to dissonance and burnout. The study concludes that reduced brain activity in empathy-related regions correlates with higher burnout severity. Notably, this reduction is also linked to higher self-reported empathy, emotional dissonance, and alexithymia. This paradox-high, self-reported empathy but low neural response-suggests that individuals vulnerable to burnout may be cognitively aware of others’ emotions but struggle to process their emotional reactions, possibly due to emotional suppression or poor regulation.

Alexithymia emerges as a consistent statistical bridge between neural blunting and subjective burnout [144]. Many works linked alexithymia to hypo-activity of AI and ACC regions that encode the precision of bodily-state prediction errors and broadcast them to prefrontal control systems [145,146]. In predictive-coding terms, repeated occupational pressure to display appropriate emotions may train top-down priors that down-weight interoceptive signals; the ensuing mismatch between expressed and felt states manifests phenomenologically as emotional dissonance and physiologically as dampened salience-network responses.

Stress physiology appears to amplify the cycle. An experimental study showed that larger cortisol reactions predict weaker empathic BOLD responses to others’ pain [147]. Chronic hypothalamic–pituitary–adrenal activation may perpetuate the insular down-regulation seen here, reinforcing alexithymia and burnout in a feed-forward loop.

Across large behavioural cohorts, higher cognitive empathy often associates with lower burnout, whereas affective over-identification predicts distress [148,149]. The current paradox-high self-reported empathy but low neural resonance-suggests that professionals compensate for blunted affective resonance with “cold” perspective-taking, preserving patient care at the cost of internal dysregulation. Reduced TPJ recruitment may further erode self/other boundaries, fostering confusion rather than compassion.

### 4.9. Impaired Resting-State Functional Connectivity

In the study [36], regarding resting-state functional connectivity, the burnout group showed weaker connectivity from the amygdala to the mPFC, dlPFC, and motor cortex. Conversely, they showed stronger connectivity between the amygdala, the insula (bilaterally), and the cerebellum (especially the vermis and anterior regions). Further analysis revealed that stronger connectivity between the amygdala and ACC was associated with a greater ability to downregulate negative emotion. This supports the hypothesis that impaired amygdala–ACC communication may underlie emotion regulation deficits [150,151]. Stress severity (MBI-GS scores) correlated positively with connectivity between the left amygdala and the insula and hypothalamic-thalamic region, indicating heightened salience and arousal signaling in more stressed individuals.

No significant group differences in cortisol levels were observed at any of the seven sampling points throughout the day. This supports conflicting findings about cortisol in burnout and suggests that neural markers might be more sensitive indicators of stress-related dysfunction. The findings indicate a functional disconnection in emotion-regulating circuits in individuals suffering from long-term work-related stress, especially affecting the amygdala’s regulatory coupling with the mPFC and ACC [128]. The strengthened connectivity with the insula and cerebellum may represent compensatory or maladaptive mechanisms. Notably, the cerebellum, typically involved in motor functions [152], is also implicated in emotional and stress processing [153,154,155] and may play a role in HPA axis regulation via its connections with the hypothalamus [156,157]. The study concludes that burnout is characterized by a specific impairment in down-regulating negative emotions, not a general inability to regulate emotions. This dysfunction may increase vulnerability to depressive symptoms and explain the comorbidity between chronic stress and mood disorders.

### 4.10. Excessive DLPFC Activity and Reduced Neural Efficiency

The pediatric-resident data [37] show that individuals who report high emotional exhaustion + depersonalization recruit the right dorsolateral prefrontal cortex (DLPFC) more strongly than their non-burned-out peers to meet the basic attentional demands of a Stroop task. In everyday clinical work, this pattern is best understood as a sign of reduced neural efficiency rather than superior effort: the DLPFC must “push harder” to preserve performance that healthy circuits can achieve with less metabolic cost. Basic neurobiology explains why. Acute and chronic stress drive surges of catecholamines (noradrenaline, dopamine) in prefrontal networks; when those surges are prolonged, they weaken the recurrent synaptic activity that typically underpins working memory and top-down control, forcing the cortex to compensate with additional firing and broader recruitment [158].

Stress hormones do not act in isolation. Repeated activation of the hypothalamic-pituitary-adrenal (HPA) axis keeps cortisol tonically elevated. It flattens its diurnal slope-an endocrine signature repeatedly linked to slower processing speed and poorer executive performance in large cohort meta-analyses [159]. Cortisol excess also amplifies limbic input to the prefrontal cortex, creating an internal milieu in which emotionally salient distractions more readily capture attention. Clinically, this maps onto the residents’ subjective depersonalization (emotional distancing as a coping strategy) and the concentration lapses many physicians report after a string of overnight or high-acuity shifts.

A second, complementary mechanism is stress-related neuroinflammation. Chronic psychological stress is now known to upregulate peripheral and central cytokines (e.g., IL-6, TNF-α); these cytokines cross or compromise the blood–brain barrier, alter glial regulation of glutamate, and further disrupt prefrontal signal-to-noise ratios [160]. In practical terms, an inflamed, catecholamine-saturated DLPFC resembles a “noisy” control tower: it can still direct traffic but with slower, more error-prone guidance.

The downstream behavioural manifestation is decision fatigue—the measurable decline in judgement accuracy and risk calibration that accumulates over a duty period. A recent systematic review across healthcare settings confirms that decision fatigue increases the likelihood of diagnostic slips and procedural errors, especially during high-complexity tasks and circadian lows [161]. The fMRI finding of heightened DLPFC effort during conflict monitoring provides a neural substrate for that phenomenon.

### 4.11. Reduced DLPFC Activation During Cognitive Demands

The long-term sick leave (LTSL) group in [38] showed a striking absence of dorsolateral prefrontal cortex (DLPFC) recruitment during the 2-back working-memory challenge, even though accuracy was preserved. To understand what that silent DLPFC signal means, it helps to place it against the background of other disorders and experimental pathophysiologies mapped with fMRI.

In healthy volunteers, a single episode of psychosocial stress rapidly blunts DLPFC BOLD responses on n-back tasks; catecholamine and glucocorticoid surges gate down delay-cell firing within minutes, yet behaviour is only modestly slowed. Qin and colleagues’ seminal “cold-pressor” study canonically demonstrates this low-gain state [162]. The burnout pattern looks like a chronic perpetuation of that same neuromodulatory switch.

Functional imaging in post-traumatic stress disorder repeatedly shows dorsal-executive hypofrontality during working memory or cognitive reappraisal, paired with hyper-salience in amygdala and mid-cingulate nodes [163]. The neural echo of trauma, therefore, mirrors the burnout profile, but with an additional emotional-threat bias that the LTSL participants did not exhibit, underscoring how pure occupational overload can disengage DLPFC without mobilising limbic alarm circuits.

Meta-analyses and reviews in attention-deficit/hyperactivity disorder report under-activation of the right (and often bilateral) DLPFC across attention and working-memory paradigms, reflecting maturational lag and dopaminergic hypofunction rather than acquired stress damage [164,165]. That hypo-frontality is present from childhood and is typically accompanied by reduced accuracy, highlighting that the adult burnout signature is an acquired, reversible low-gain state rather than a developmental ceiling.

The muted DLPFC response in burnout is best interpreted as a low-gain, high-allostatic-load state: prolonged cortisol and catecholamine exposure keep the executive network in “idling” mode, curbing metabolic cost but slowing response speed. Because the posterior memory circuits remain intact and accuracy is spared, the data point to a functional gating mechanism rather than irreversible circuitry loss. This places work-stress exhaustion on a continuum with other stress-linked conditions yet distinguishes it from disorders where DLPFC dysfunction arises from developmental delay, glutamatergic dysconnectivity, or neuroinflammatory injury.

### 4.12. No Changes in the Hippocampus

Despite cognitive deficits and HPA abnormalities, hippocampal volumes—including subfields CA1 and CA2/3—did not differ between groups [39]. This suggests that cognitive dysfunction is likely functional rather than structural in origin, or that hippocampal atrophy has not yet developed in this patient population.

### 4.13. Connectomic Disintegration in Burnout

The whole-brain graph profile seen in burnout [40]-longer characteristic path length, higher λ, and lower global efficiency-maps onto the “de-integrated” connectome already described in late-life depression, where reduced worldwide efficiency and prolonged path length track the severity of affective and cognitive slowing [166]. Post-traumatic stress disorder shows a complementary pattern of topological disruption, with altered path length and efficiency that likewise betrays a loss of the normal small-world balance between segregation and integration, underlining that chronic stress states of different origins converge on a common failure of economic information transfer [167]. Clinically, such global detours help explain why individuals with burnout report thought fatigue and difficulty orchestrating complex tasks: messages must traverse extra relay nodes before pre-frontal hubs can coordinate a response.

Against this background of slowed global traffic, specific sensory nodes in burnout become locally dominant. The bilateral cuneus, superior occipital gyrus, and fusiform gyrus act as high-throughput “shortcut routers”, mirroring the visual-system hyperactivation seen when people at risk for severe postpartum mood episodes engage working-memory and emotion tasks [168] and the stress-induced amygdala–fusiform coupling that prioritises emotionally salient faces after acute psychosocial challenge [169]. In longitudinal PTSD cohorts, remission is marked by the restoration of hippocampus V4–V4 connectivity. In contrast, persistence is marked by its absence, underscoring that hyper-responsive occipital hubs become maladaptive when they are no longer anchored to mnemonic context [170].

Indeed, the network-based-statistic sub-network isolated here shows that most severed effective edges originate in these visual hubs and terminate in the right hippocampus. Stress research ties such disconnection to glucocorticoid dynamics: childhood trauma blunts basal cortisol yet increases prefrontal–extrastriate coupling at the expense of prefrontal–hippocampal links. This arrangement predicts over-general autobiographical memory and emotional dysregulation [171]. Experimental work during an MRI stressor further confirms that shifts in hippocampal connectivity-strengthened ties to hypothalamic stress circuits and weakened ties to regulatory cortices-track moment-to-moment feelings of being “stressed” [172]. The burnout pattern, therefore, reads as a chronic, cortico-visual imprint of allostatic load in which sensory evidence floods the system while hippocampal gating is offline.

A second compensatory pivot appears in the right superior temporal gyrus, whose betweenness centrality rises in burnout and whose connectivity with insula and dorsolateral prefrontal cortex scales with repetitive negative inner speech in major depression [173]; therapeutic electro-convulsive treatment reduces the same STG centrality alongside rumination scores, hinting that this auditory–language node becomes a relay for self-referential chatter when higher-order control falters [174]. Conversely, the left anterior cingulate cortex loses nodal efficiency, echoing adolescent-depression data in which an inflexible dorsal ACC predicts poor inhibitory control and earlier disorder onset [175]. Together, these shifts sketch a mechanism whereby pre-frontal governance is weakened, visual and auditory systems compensate, and limbic memory circuits fall out of synchrony-producing the emotional exhaustion, depersonalisation, and diminished efficacy that define burnout’s phenotype.

### 4.14. Hierarchical Connectome Disruptions and Transcriptomic Drivers in Occupational Burnout

The expansion of manifold eccentricity in somatomotor and visual cortices suggests that large swaths of the sensorimotor hierarchy have become more segregated from the transmodal apex. Similar—but not identical—hierarchical distortions are now reported in several stress-related or affective conditions. In major depressive disorder (MDD), both hemispheres show compression of the principal gradient and abnormal left–right asymmetry that predicts Beck Depression Inventory scores [176]. In contrast, post-traumatic stress disorder (PTSD) is characterised by an increased gradient range anchored in visual and salience hubs that scales with Clinician-Administered PTSD Scale severity [177]. By contrast, the burnout pattern is dominated by regional expansion (not compression). It is weighted toward unimodal networks, mirroring the “perceptual overload” and psychomotor fatigue clinicians recognise in exhausted shift workers.

Gradient-linked genes that were down-regulated (PLS^−^) are enriched for synaptic vesicle trafficking, GABA/glutamate release machinery, and metal-ion binding. A growing body of work indicates that chronic psychosocial or restraint stress in rodents drives an excitatory/inhibitory (E/I) shift and pre-synaptic vesicle depletion, partly via zinc and copper dys-homeostasis in corticolimbic synapses [178,179]. Parallel human data show that iron, manganese, and copper imbalance potentiate oxidative stress and neurotransmission failure across neuropsychiatric spectra, including depression and neurodegenerative disorders [180,181]. Thus, the PLS^−^ signal in burnout likely captures a broader “metallomic-synaptic” axis of vulnerability that could be tractable with nutritional or chelation strategies under investigation in mood and neurodegenerative trials.

Cell-type enrichment pointed to astrocyte transcripts among PLS^−^ genes. Astrocytes regulate extracellular potassium and glutamate, set synaptic gain, and buffer divalent metals. Recent reviews highlight astrocyte–synapse structural plasticity as a unifying mechanism linking traumatic stress, substance use, and neurodegeneration [182]. Burnout may therefore join a class of “astro-gliopathies” in which glial metabolic fatigue and impaired ion buffering drive network desegregation and the observed gradient expansion. Astrocytic chloride transport and sleep-dependent glymphatic clearance are now recognised as levers of the cortical E/I ratio; their disruption can prolong wake-like cortical states and erode functional hierarchies.

PLS^+^ genes were enriched for core clock components (e.g., PER1, ARNTL), and shift-work studies in healthcare personnel show that even a week of night shifts attenuates fronto-parietal connectivity and degrades overnight glymphatic clearance [183]. Neuroimaging in healthy adults reveals circadian oscillations in cortical thickness and steroid hormones, with misalignment blunting those rhythms and shrinking default-mode hubs [184]. Moreover, large-scale fMRI demonstrates that circadian and ultradian modulation of whole-brain activity is diminished in pathological tissue [185]. The burnout transcriptomic signature dovetails with clinical data: chronic clock disruption in rotating-shift nurses may downregulate astrocytic and synaptic maintenance genes, precipitating hierarchical desegregation.

## 5. Appeal to Policymakers

Burnout can no longer be dismissed as a passing state of exhaustion or simple job dissatisfaction. Modern neuroimaging repeatedly shows that chronic occupational stress leaves unmistakable fingerprints on the brain. Core emotion-processing hubs such as the amygdala enlarge, especially in women, heightening reactivity to threat; executive regions of the prefrontal cortex thin and disconnect, blunting cognitive control and emotional regulation; the caudate and putamen shrink, slowing thought and action; and the intricate “rich-club” highways that keep distant brain areas in sync lose their efficiency. These changes translate directly into the problems employers and society already observe: poorer clinical judgement, slower decision-making, precarious emotional balance, and a steep rise in preventable errors. Crucially, the neurobiology of burnout diverges from that of depression or post-traumatic stress, confirming that it is a distinct medical condition rather than a variant of another disorder.

Because these alterations fulfil every conservative criterion for disease—objective, reproducible, and disabling—policymakers must move burnout out of the grey zone of “occupational phenomenon” and into the family of recognised mental illnesses. Doing so would unlock parity of insurance coverage for evidence-based therapies, oblige employers to respect safe staffing ratios and maximum shift lengths, and open dedicated research lines for biomarker-guided prevention. Official classification would also permit national health registries to track incidence, costs, and treatment outcomes, which is impossible while the diagnosis floats outside formal nosology. And because men and women manifest different neural vulnerabilities-amygdala hypertrophy is far more common in women, caudate loss in men-legislation must be gender-responsive, ensuring that preventive guidelines, screening intervals, and rehabilitation programmes account for biological sex.

The economic argument is equally compelling. Untreated burnout drives months-long sick leave, silent presenteeism, and downstream liability from safety-critical errors. Early treatment halves average absence duration and reduces on-the-job mistakes, returning measurable savings to public budgets and private balance sheets. Workforce retention improves, gender gaps in long-term disability shrink, and children grow up with less stressed caregivers—a ripple of inter-generational benefit that will never appear on a short-term ledger yet shapes future public-health expenditure.

Legislators, therefore, face a clear choice: preserve the status quo and continue shouldering escalating productivity losses, or acknowledge that burnout is a genuine brain-based illness, deserving of the same statutory protections already afforded to depression or anxiety. The science is firm, the societal stakes are high, and the moral imperative is undeniable. Recognising burnout as a mental disorder, funding its treatment, and embedding prevention into labour laws are no longer merely options—they are a duty owed to workers, patients, the economy, and the generations that follow.

## 6. Interventions That Can Reverse the Neural Changes in Burnout

**Figure 2:**
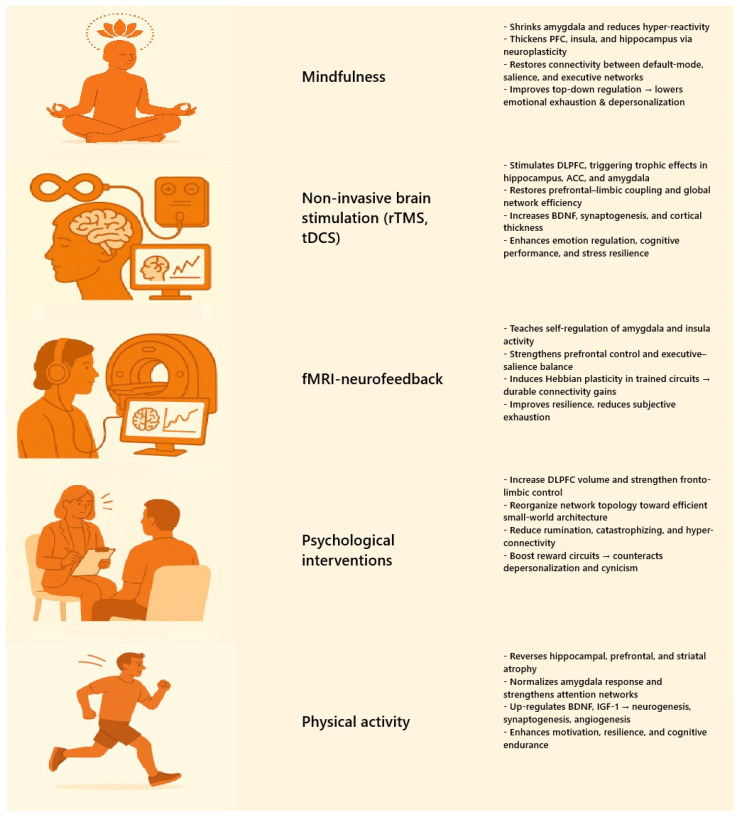
Brain-targeting interventions that may help treat burnout.

Growing evidence shows that structural and functional abnormalities—including amygdala hypertrophy, ventro-/dorsolateral prefrontal cortex (PFC) thinning, caudate atrophy, and network disintegration—are plastic rather than permanent. Carefully designed behavioural programs can normalise many of these markers, often in parallel with clinical recovery. Below are the best-supported approaches to date. The possible mechanisms of action of individual interventions are presented in Figure 2.

### 6.1. Mindfulness

Magnetic-resonance studies of occupational burnout converge on a characteristic neural profile: limbic alarm centres such as the amygdala and posterior cingulate become hypertrophic and hyper-reactive, prefrontal regulatory hubs (dorsolateral and ventromedial PFC) lose cortical thickness and metabolic efficiency, the striatal caudate and putamen shrink-with knock-on effects on motivation-and long-range network integration through rich-club connections and the default-mode/salience bridge progressively deteriorates. These lesions explain why exhausted clinicians and nurses show heightened emotional volatility and poorer executive control during demanding tasks. Mindfulness-based interventions appear to drive neuroplasticity in precisely the opposite direction. Many reviews have demonstrated the effectiveness of mindfulness in reducing burnout symptoms. For example, a meta-analysis by Fendel et al. found that mindfulness reduces physician burnout and stress [186]. Another meta-analysis revealed that MBI was more effective than passive comparators in reducing psychological distress, anxiety, depression, and burnout-personal accomplishment among nurses [187]. A 2020 study demonstrated that mindfulness training reduces burnout, producing lower scores for emotional exhaustion and depersonalization and higher scores for personal accomplishment [188]. Moreover, the effects of meditation are durable—after 6 months, the mindfulness groups were shown to have significantly better results than the control groups in reducing and preventing burnout and stress [189].

This meditation practice has been shown to induce neuroplastic changes, for example, by increasing BDNF production [190] and engaging other, complex mechanisms [191]. There is ample evidence from peer-reviewed studies that mindfulness induces structural and functional brain reorganization, as demonstrated by MRI neuroimaging.

Long-term meditators and participants in eight-week Mindfulness-Based Stress Reduction (MBSR) programmes show significant grey-matter expansion in the hippocampus, posterior cingulate cortex, the temporo-parietal junction, and the cerebellum [192] together with measurable thickening of the PFC and insula [193]; these sites overlap the very regions that atrophy in burnout. Parallel longitudinal work demonstrates that brief mindfulness practice can shrink the amygdala and dampen its basal coupling with stress-responsive cingulate cortices, effectively reversing the limbic hypertrophy reported in female exhaustion syndrome [194,195]. A five-week mindfulness course has also been linked to restoration of normal caudate volume, suggesting recovery of striatal circuits that erode under chronic workload pressure [196].

Functional imaging tells a complementary story. Just one month of mindfulness training strengthens interconnectivity among the default-mode, salience, and central-executive networks, re-establishing top-down regulatory links that have been shown to fragment in longitudinal burnout cohorts [197,198]. In parallel, targeted analyses of the amygdala–subgenual ACC pathway reveal rapid reductions in stress-related functional coupling after mindfulness practice, mirroring the failure of down-regulation (and the dorsal ACC “inflexibility”) observed when burned-out employees face repeated psychosocial stressors [195].

At the systems level, graph-theoretical work indicates that mindfulness therapy shortens characteristic path length, increases global efficiency, and facilitates rest-to-task reconfiguration across executive, salience, and default-mode circuits-precisely the network metrics that worsen as burnout deepens [199,200,201]. Where burnout studies document impoverished mid- and long-range connectivity, mindfulness induces a more integrated, small-world topology, suggesting a wholesale repair of information flow from sensory cortex through limbic gatekeepers to prefrontal control nodes.

Clinically, these circuit-level changes accompany-and plausibly mediate-reductions in emotional exhaustion and depersonalisation reported by health-care workers who complete mindfulness programmes while normalising attentional performance on Stroop-like tasks. Taken together, multi-database MRI evidence supports the view that mindfulness does not merely buffer subjective stress but actively counteracts and may even reverse the structural and functional brain alterations that underlie burnout.

For mindfulness practice to be beneficial in alleviating burnout symptoms, we recommend implementing protocols that involve at least eight weeks of regular sessions, ideally twice weekly. Furthermore, future studies should examine the neural impact of mindfulness on individuals with burnout using structural and functional MRI. This will build a neuroimaging evidence base that mindfulness leads to beneficial and lasting neuroplastic changes in burnout.

### 6.2. Physical Activity

The earliest synthesis to focus exclusively on physical activity (PA) and burnout collated ten longitudinal or intervention trials and found a convergent inverse association: every study that measured moderate-to-vigorous PA reported lower subsequent emotional exhaustion, with strong evidence for longitudinal designs and moderate evidence for interventions. The authors concluded that “regular PA constitutes a promising medium for burnout prevention,” while calling for larger RCTs to address residual bias factors [202]. Subsequent meta-analysis of six randomized exercise trials (n = 248) confirmed the trend but highlighted the field’s infancy. The pooled standardized mean difference favoured exercise (SMD = −0.16) yet the 95% CI crossed zero (−0.41 to +0.09), a limitation driven by heterogeneous protocols (aerobic vs. yoga, 4–16 weeks) and small sample sizes. Importantly, statistical heterogeneity was nil (I^2^ = 0%), underscoring that all trials moved in the same protective direction even if power was insufficient for a definitive estimate [203]. A 2024 review confined to physicians, nurses, and allied professionals and retained 21 studies. The results showed that meeting the WHO guideline of moderate activity predicted lower future burnout, especially for emotional exhaustion and depersonalization [204].

As we know, chronic occupational stress thins the ventromedial and dorsolateral prefrontal cortex, shrinks striatal structures such as the caudate, enlarges or hyper-sensitises the amygdala, and, at the network level, weakens long-range “rich-club” connectivity-precisely the pattern reported across the MRI studies on burnout. Physical activity counteracts each of these lesions along the same anatomical axes. In late-life adults who walked briskly three times a week for a year, the anterior hippocampus grew by about two per cent, whereas matched controls lost volume; memory improved in parallel, showing that aerobic training can reverse stress-linked limbic atrophy rather than merely slowing it [205]. Six months of similar aerobic training in another randomised trial produced gray- and white-matter expansion in the anterior cingulate and lateral prefrontal cortex—regions whose cortical thinning marks emotional exhaustion and depersonalisation—while stretching–toning controls showed no change, confirming that activity restores prefrontal substrate instead of simply maintaining it [206]. Cardiorespiratory fitness is also positively related to the volume of dopamine-rich basal ganglia: in 179 older adults, higher VO_2_max predicted larger caudate nuclei, and caudate size partly mediated the link between fitness and cognitive flexibility, the very function that fatigued residents struggle to preserve [207].

Beyond structure, even a single bout of running can recalibrate hyper-reactive limbic circuits: a crossover fMRI study showed that twelve minutes on a treadmill shifted the amygdala’s response away from threat and towards positive faces and tightened its coupling with orbitofrontal cortex, an effect most substantial in habitually active participants, implying that regular exercise “pre-conditions” this stress hub to remain quiet [208]. At the systems level, moderate-intensity cycling enhances functional connectivity inside the dorsal attention network. It strengthens its dialogue with the default-mode network, precisely the long-range links fragmenting when burnout weakens the rich-club organisation. In contrast, high-intensity exercise does not, mirroring the inverted-U principle that moderate arousal best supports integrative brain function [209].

Mechanistically, aerobic and resistance work up-regulate brain-derived neurotrophic factor [210], insulin-like growth factor-1 [211] and vascular growth factors [212], promoting neurogenesis in hippocampus [213], synaptogenesis in prefrontal cortex [214] and striatum [207], angiogenesis throughout, and lowering systemic inflammation [215]-biochemical changes that map onto the very circuits eroded by chronic stress. Thus, prescribed physical activity is not merely a behavioural antidote to burnout but a biologically grounded strategy capable of re-growing lost gray matter, normalising limbic excitability, and reconnecting the brain’s communication backbone.

### 6.3. Psychological Interventions

Another possible treatment option for burnout that can reverse functional and structural changes in the brain is broadly defined psychological interventions. Numerous reviews have demonstrated the potential of cognitive behavioral therapy (CBT) and related procedures to induce functional reorganization [216,217,218,219,220,221,222,223,224,225]. An 11-week CBT programme for chronic pain increased grey-matter volume in the dorsolateral prefrontal cortex (DLPFC) and adjacent sensory areas; growth in DLPFC volume tightly paralleled reductions in catastrophising, a cognitive style that overlaps phenomenologically with burnout rumination [226]. A six-month course of CBT for psychosis went a step further, reorganising functional connectivity so that the DLPFC exerted stronger top-down control over the amygdala and other limbic nodes; the size of this connectivity gain predicted symptom remission eight years later, underscoring that talk therapy can leave a durable anatomical signature in the very fronto-limbic circuit whose inefficiency has been documented in burned-out physicians during reasoning tasks [227].

Where mindfulness and CBT converge on executive and limbic repair, Acceptance-and-Commitment Therapy (ACT) appears to recalibrate whole-brain communication. Network-based fMRI analyses in chronic-stress pain patients showed that ACT reduced the pathological hyper-connectivity linking the default-mode, fronto-parietal, and salience networks and restored a more energy-efficient small-world topology—essentially the mirror image of the rich-club fragmentation described in longitudinal burnout cohorts [228].

Training compassion rather than attention adds a reward-system dimension that may offset the cynicism and emotional blunting of depersonalisation: eight weeks of compassion cultivation selectively boosted activity in ventral striatum, pre-genual anterior cingulate, and medial orbitofrontal cortex while reversing empathy-induced distress, indicating a shift from aversive arousal to affiliative motivation [229].

The advantages of this type of intervention include its relatively low cost and widespread availability. Disadvantages include the potential difficulty of finding a therapist specializing in burnout therapy and the need for long-term treatment to observe positive effects. We recommend studying the effectiveness of psychological interventions for burnout over a long period, for example, in a six-month protocol with two weekly therapy sessions. Furthermore, studies confirming the neural impact of psychological interventions for burnout using MRI are necessary.

### 6.4. fMRI-Neurofeedback

Real-time fMRI neurofeedback (rtfMRI-NF) offers a direct way to teach people how to see and steer circuits that collapse under chronic occupational stress. In burnout, those circuits are skewed toward limbic alarm (amygdala, anterior insula, salience network) and away from executive oversight (dorsolateral and ventromedial prefrontal cortex, executive-control network). Proof-of-concept studies now show that individuals can learn-often within a handful of sessions-to rebalance those systems, with measurable downstream gains in resilience and mood.

The most explicit network-level demonstration comes from Krause and colleagues, who trained volunteers to shift the difference signal between the salience and executive-control networks. Across three sessions, participants learned to tilt activation toward the executive network. They could still do so under an acute stressor, indicating transfer of the skill to a real-life threat context [230]. Because an inflated salience-over-executive ratio is one of the best functional fingerprints of physician and nurse burnout, mastering this “network dial” could, in principle, restore the dynamic resource allocation that falters during emotional exhaustion.

At the nodal level, a rich body of work shows that the amygdala—the structure most consistently hypertrophied or hyper-reactive in burned-out women—can be down-regulated voluntarily. In combat veterans, amygdala-focused rtfMRI-NF reduced amygdala activity during training and normalised resting-state connectivity with ventrolateral PFC and dorsal ACC; the larger the connectivity gain, the greater the symptom relief [231]. A recent double-blind trial replicated clinical benefits, confirming that participants can acquire durable control over amygdala output [232].

The insula, whose thinning in nurses tracks emotional exhaustion, is also trainable: a 2025 systematic review covering 25 rtfMRI-NF studies concluded that insula self-regulation is “efficient” and that symptom improvements persist after training ends, albeit with room for protocol optimisation [233]. Because the anterior insula drives interoceptive distress signals that feed burnout’s feeling of being “spent,” the capacity to quiet this hub could directly ease subjective exhaustion.

Although burnout-specific rtfMRI-NF trials are only beginning, a pilot with surgical residents is encouraging: eight weeks of neurofeedback (delivered with EEG but guided by the same self-regulation principles) reduced cognitive workload and log-transformed EEG indices of stress, suggesting a shift toward more efficient neural processing during demanding tasks [234]. Integrating rtfMRI-based targets into such occupational programmes could sharpen their anatomical precision-e.g., teaching residents to up-regulate dorsal PFC while down-tuning amygdala during simulated code blues.

Mechanistically, repeated successful self-regulation is thought to induce Hebbian-like plasticity in the trained circuit: strengthening top-down PFC synapses, pruning hyperactive limbic afferents, and gradually normalising whole-brain connectivity. Longitudinal work in affective disorders already shows that gains in connectivity integrity after rtfMRI-NF can persist for months, and grey-matter increases in the ACC and dorsomedial PFC have been reported after as few as 15 sessions in anxiety populations [235]. Burnout shares the same prefrontal-limbic bottlenecks, so these findings imply a realistic path toward anatomical reversal rather than mere symptom management.

The advantages of fMRI neurofeedback include its undoubted effectiveness, long-lasting effects, the relatively small number of interventions required to achieve positive results, the ability to target altered brain activity directly, and the complete personalization of interventions. The disadvantages include the lack of research confirming the effectiveness of fMRI neurofeedback in the treatment of burnout, the limited availability of this procedure (equipment is usually available in specialized research centers), and the high cost of treatment.

### 6.5. Non-Invasive Brain Stimulation

The same fronto-limbic and striatal circuits that thin, swell, or drift apart in MRI studies of burnout have already proved plastic under non-invasive brain-stimulation (NIBS) protocols. In treatment-resistant depression—a condition that shares prefrontal erosion with occupational exhaustion—ten weekdays of 20 Hz rTMS (repetetive transcranial magnetic stimulation) over the left DLPFC enlarged the ipsilateral hippocampus by 3.4%, showing that stimulation of a superficial executive hub can trigger trophic growth deep in the limbic system, a remote effect mediated by cingulum projections [236]. A longitudinal study that scanned patients twice over four weeks of standard high-frequency rTMS found that responders exhibited measurable thickening of the rostral anterior cingulate and lateral orbitofrontal cortices [237].

Beyond local grey-matter gain, intensive stimulation protocols remodel whole-brain communication. Stanford Neuromodulation Therapy-an accelerated, connectivity-guided intermittent theta-burst schedule delivering fifty sessions in five days-increased the anticorrelation between the individually targeted left DLPFC and the default-mode network, effectively tipping the see-saw back toward task-positive executive control; that shift persisted one month later [238]. When stimulation is aimed with functional-connectivity maps that maximise overlap between the DLPFC and its white-matter tract to the amygdala, a week of 5 Hz rTMS dampens amygdala reactivity during emotional challenges. It strengthens resting DLPFC-amygdala coupling, demonstrating that top-down regulation of the limbic “alarm bell” can be reinstated non-pharmacologically [239]. Converging graph-theoretical work shows that ten sessions of 10 Hz rTMS raise voxel-wise degree centrality in the anterior cingulate and medial frontal hubs—essentially restoring the global integration and hub status that crumbles when chronic stress drives rich-club networks toward a more regular, less efficient topology [240].

Transcranial direct-current stimulation (tDCS) yields complementary evidence. In the first double-blind pilot with clinically burned-out employees, fifteen anodal sessions over the left DLPFC produced larger gains in sustained attention and executive updating than sham, indicating functional rescue of the circuits whose grey-matter loss undermines cognitive efficiency in burnout [241]. Multifold tDCS montages that target the frontopolar cortex likewise shift network balance: a single 20 min dose reduced anterior-insula coupling to the default-mode and salience networks while boosting frontoparietal links [242].

Mechanistically, both magnetic pulses and weak direct currents induce long-term-potentiation-like increases in brain-derived neurotrophic factor, spur dendritic spine formation and angiogenesis around the stimulation site, and propagate activity-dependent trophic signals along connecting tracts, explaining how hippocampus, caudate, or amygdala—all distant from the coil or electrode—can show downstream volumetric change. Clinically, the translation is straightforward: targeting the left DLPFC (Brodmann 9/46) with 20–30 sessions of 10 Hz rTMS or 600-pulse iTBS, or delivering 2 mA anodal tDCS for 20 min across fifteen workdays, offers a biologically grounded route to regrow atrophied executive cortex, quiet a hyper-reactive amygdala, and re-stitch long-range network fibres—precisely the anatomical repair job that MRI has shown that burnout needs.

The advantages of these methods include safety, moderately low intervention costs, and a growing evidence base regarding the general mechanisms of action of brain stimulation in the treatment of many neurological and psychiatric disorders. Disadvantages include limited availability, expensive equipment (for rTMS), and the lack of a solid evidence base from clinical trials confirming the effectiveness of NIBS in burnout.

### 6.6. Prevention Is Better than Cure

The interventions outlined above have the potential to help treat burnout. However, it’s easier to implement preventative measures than to treat the profound brain and emotional changes it causes. Emerging evidence indicates that psychologically informed interventions grounded in mindfulness and physical activity can be scalable, primary prevention strategies against occupational burnout, particularly in health-care and other high-strain settings. Multiple systematic reviews and meta-analyses of MBIs—including standardized programs such as MBSR and brief workplace-adapted formats—show small-to-moderate short-term reductions in burnout (most consistently on emotional exhaustion), alongside gains in well-being, resilience, and perceived stress, with benefits sometimes persisting to medium-term follow-up despite heterogeneity in trial quality and delivery (in-person vs. Digital) [243,244,245].

Unfortunately, there are no guidelines on when to implement interventions to prevent burnout. The first approach is to prevent the onset of symptoms. Employees in high-risk occupations should receive ongoing psychological support, participate in sports, and consider participating in regular mindfulness practices. This will allow them to effectively cope with their job duties’ cognitive and behavioral demands. The second approach is implementing therapeutic interventions when the first signs of burnout are detected. This approach carries a particular risk that the individual will downplay the symptoms or fail to connect them with burnout, as not every employee is aware of or familiar with this condition.

Future studies should use MRI to track the impact of preventive interventions on the brains of individuals at risk for burnout and compare the results with those of individuals who do not participate in any interventions. This will help uncover the neural and protective mechanisms triggered by each intervention.

Educational institutions should promote knowledge about burnout and its devastating consequences. Physicians should ensure they have the knowledge needed to diagnose burnout and recommend the interventions mentioned above to patients, which can be helpful in treatment.

## 7. Open Questions

### 7.1. Causality and Temporal Dynamics

Single-scan, cross-sectional snapshots still dominate the neuroimaging literature on burnout. Those slices of time can reveal associations—a thinner ventromedial prefrontal cortex (vmPFC) here, an enlarged amygdala there—but they leave the field guessing about the proper direction of influence. Does chronic overload sculpt the brain, or do pre-existing neural traits predispose someone to buckle under pressure? At present, the answer is opaque because almost every dataset enrolls people after exhaustion is already entrenched.

A proper causal map demands three methodological upgrades. First, prospective, dense-sampling cohorts must begin before individuals enter high-risk environments (for example, the first day of medical school, nursing training, or onboarding in call-center work). By repeating structural and functional scans every few months—alongside ecological stress diaries, wearable actigraphy, and salivary cortisol—researchers can plot individual trajectories: who accumulates neural wear-and-tear, who recovers, and who remains resilient despite escalating demands.

Second, we require randomized stress-modulation experiments. Ethical stress induction is off the table, but workload-reduction, sleep-extension, or mindfulness programs are randomizable. Embedding imaging before and after such interventions lets us test whether easing strain reverses the circuits that appear damaged in cross-sectional work (e.g., striatal atrophy or rich-club disintegration). Suppose neural recovery parallels symptom relief; that argues strongly for a causal stress-brain pathway. If amygdala size or vmPFC thickness hardly budges, those alterations might represent pre-existing risk factors or slow-healing scars.

Third, causality hinges on fine-grained temporal resolution during acute challenges. Study [8] hints that, under repetitive psychosocial pressure, healthy controls gradually dial down dorsal ACC reactivity, whereas burned-out workers ramp it up-an early sign of regulatory failure. Yet this insight rests on coarse block-design averages. Future work should pair event-related fMRI with second-by-second autonomic indices (pupil dilation, electrodermal activity) and computational tools like hidden Markov modelling to expose the microdynamics of collapse. Such approaches can reveal whether burnout brains enter maladaptive “high-cost control” states sooner, stay stuck longer, or exit less completely after the stressor ends.

Temporal questions extend beyond the lab. Only two papers ([4,6]) provide accurate longitudinal imaging, and even they capture just a single post-rehabilitation scan roughly 12–18 months later. From those limited data, we learn that prefrontal cortex thickness and caudate volume can rebound, while amygdala enlargement lingers-yet the critical when and how fast remain a blur. High-frequency follow-ups—monthly for the first half-year of sick leave, quarterly thereafter—are essential to chart the half-lives of neural lesions and to determine whether prolonged abnormalities mark latent vulnerability to relapse.

Finally, temporal dynamics matter for prediction. Cross-sectional correlates seldom translate into actionable risk scores, but slopes and inflection points might. A nurse whose rich-club efficiency drops 5% in three months could be flagged for early intervention long before self-reported exhaustion peaks. Achieving that vision will require federated, multi-center consortia using harmonized pipelines and Bayesian growth-curve modelling to distinguish signal from site noise.

In short, until neuroimaging moves from static portraits to cinematic storytelling—capturing brains before, during, and after burnout—causal claims will remain tentative, and prevention strategies will continue to fly blind.

### 7.2. Sex Differences and Hormonal Influences

The neuroimaging evidence gathered so far draws a vivid but still enigmatic picture: men and women do not share the same neural fate under chronic occupational stress. In sample after sample, female professionals show enlarged amygdalae, thinner left superior temporal and frontal cortices, and measurable declines in attention and memory. Meanwhile, male counterparts more often present with caudate atrophy and preserved cognition. These divergent footprints cry out for an explanation, yet the field remains largely speculative. Researchers routinely cite estrogen-driven glutamatergic plasticity or testosterone effects on the striatum. Still, almost no human study has measured estradiol, progesterone, testosterone, or their metabolites during scanning. No one combined imaging with in vivo assays of sex-steroid receptors or downstream gene expression. Life-course stages that remodel hormone signalling—puberty, pregnancy, postpartum, perimenopause, and andropause—have been left unexplored, and even the menstrual cycle is usually ignored; scan timing rarely distinguishes follicular from luteal phase, and oral-contraceptive use is lumped in or quietly omitted. The result is a literature rich in descriptive sex contrasts but poor mechanistic traction.

Methodological unevenness compounds the problem. Women dominate many burnout cohorts because they recruit nurses, while physician samples skew male; few reach balanced numbers large enough to test sex as a biological variable rather than a nuisance covariate. Subgroup sizes often fall below twenty, inflating false positives and negatives. Shift work, sleep debt, and nutritional patterns—environmental factors that differ systematically between sexes—are seldom modelled, further muddying biology with lifestyle noise.

Because the descriptive signals are so robust, the next wave of studies must move from observation to experiment. One obvious step is a menstrual cycle protocol: scan thirty naturally cycling women four times—early follicular, pre-ovulatory, mid-luteal, pre-menstrual—while measuring serum estradiol, progesterone, LH, and FSH, and matching them to a male control group scanned at identical intervals. High-resolution structural MRI, resting-state fMRI, and glutamate-sensitive MRS in the amygdala and vmPFC could reveal whether the volumetric swings seen in cross-section intersect with hormone peaks. A second design should manipulate hormones directly: randomise mid-luteal women to receive estradiol or placebo or use a GnRH-agonist protocol with low-dose add-back, then probe emotion-regulation networks with fMRI and stress-reactive cortisol assays. At a larger scale, a decade-long lifespan cohort beginning in late adolescence and spanning to older adulthood could chart how sex-specific trajectories of amygdala and striatal structure emerge, plateau, and perhaps converge later in life.

Analytically, these projects will need hierarchical linear models that treat hormone levels as time-varying covariates nested within sex, allowing investigators to disentangle within-person hormonal oscillations from between-person sex differences. Multimodal fusion techniques that marry steroid concentrations, peripheral transcriptomics, and MRI phenotypes into standard latent variables can then pinpoint causal pathways. Dynamic Bayesian networks or other causal discovery algorithms applied to dense longitudinal data may finally establish whether hormone surges drive connectivity breakdown or vice versa. Power calculations must be built around interaction effects, not main effects; otherwise, the field will remain underpowered to answer its driving question.

If these gaps are closed, the clinical dividends could be substantial. We might learn that amygdala hypertrophy spikes peri-ovulation, suggesting that scheduling adjustments or preventive leave could blunt affective overload in highly stressed women. Caudate volume is a male-specific biomarker of emerging executive fatigue, alerting occupational health teams before performance falters. Therapeutically, estradiol modulators or carefully timed hormone-replacement strategies could buffer the female prefrontal cortex against excitotoxic stress, while dopaminergic agents might better protect male striatal circuits. Until then, sex will remain the most conspicuous yet least understood dimension of burnout neurobiology-a reminder that half of the story is still missing whenever brains are averaged across genders without a second thought.

### 7.3. Converging vs. Diverging Structural Markers

A decade of burnout MRI has produced a collage of partially overlapping, partially contradictory structural findings. Specific motifs repeat often enough to feel robust-the ventromedial prefrontal cortex almost always turns up thinner, the amygdala usually larger, the caudate frequently smaller, yet when the field tries to stitch these pieces into a single pathophysiological narrative, the seams do not align. Nowhere is this tension more evident than in the hippocampus: classical stress biology predicts atrophy here, but half of the burnout papers detect no volume loss, and several find entirely normal subfields even when emotional exhaustion is severe. Methodological heterogeneity is the obvious suspect-voxel-based morphometry at 1.5 T versus subfield segmentation at 3 T, manual tracing versus automated Bayesian inference, and whole-head versus limbic-restricted masks. Still, even studies that share platforms and pipelines diverge, hinting that biological moderators (sex, duration of stress, sleep debt, inflammatory load) are confounding the picture.

The lateral prefrontal cortex illustrates a second puzzle: many cohorts report cortical thinning that scales with exhaustion, reinforcing ideas of glutamate-mediated dendritic loss, yet the most significant sample to date shows thicker LPFC in the most stressed individuals, a finding that completely flips the prevailing story. Without quantitative MRI to parse myelin and iron content or MRS to measure neurotransmitters, it is impossible to judge whether “thickening” is genuine growth, reactive gliosis, oedema, or simply registration error. Meanwhile, the striatum—especially the caudate and putamen—oscillates between shrinkage, swelling, or stasis depending on the study, even though every team agrees these nuclei are central to motivation and cognitive control. One plausible explanation is a non-linear trajectory: early burnout might provoke dopaminergic hyperactivity and transient hypertrophy, whereas prolonged exhaustion leads to synaptic pruning and volume loss, but testing that idea requires longitudinal imaging at multiple disease stages, which almost no one has attempted.

Technical factors aggravate the biological noise. Sample sizes hover in the thirties, head coils vary from eight to thirty-two channels, and preprocessing toolboxes apply subtly different bias-field corrections that can shift cortical thickness by hundreds of microns. Few groups share raw data, so direct replication is rare, and statistical thresholds swing wildly from FWE-corrected *p* < 0.05 to uncorrected *p* < 0.005, guaranteeing a mix of actual hits and false lights. Cross-site harmonisation methods such as ComBat are still the exception, not the rule. Even basic phenotype definition differs: some authors label participants “burned-out” if any Maslach subscale is high. In contrast, others require two or three elevated dimensions, effectively sampling different syndromes under the same name.

The field needs high-field, high-sample, and high-rigour designs to move past this impasse. Seven-Tesla scanners can resolve hippocampal CA1 versus CA3, dentate gyrus versus subiculum, and separate dorsal from ventral striatum-granularity that may reveal subregion-specific stress signatures hidden in coarser data. Quantitative relaxometry, magnetisation transfer, and susceptibility mapping can dissociate thickness from demyelination, oedema, and iron deposition. Above all, studies must be longitudinal: tracking residents from their first month on the wards, nurses across rotating shifts, tech workers through product-launch sprints, and scanning them every quarter will clarify whether structures first expand, then shrink, or whether individual trajectories bifurcate into “resilient” and “vulnerable” phenotypes. Harmonised pipelines, preregistered thresholds, and open raw datasets would let consortia pool thousands of brains, turning today’s conflicting fragments into a coherent structural atlas of burnout.

Until such efforts mature, the safest conclusion is that burnout does alter brain anatomy, but not in a single, linear, one-size-fits-all fashion. Instead, different circuits may follow distinct time courses; sex hormones, sleep deprivation, and inflammatory tone may tilt effects one way or another; and what looks like a contradiction in cross-section may resolve into a predictable sequence in longitudinal view. Convergence and divergence are therefore two sides of the same coin; snapshots of a dynamic process whose accurate contours will only appear when the field stops freezing the movie at one frame and starts watching the whole reel unfold.

### 7.4. Functional Network Integrity and Cognitive Efficiency

Functional MRI studies of burnout can be divided into camps that seldom talk to each other. The first camp runs task-based paradigms—clinical reasoning, Stroop, n-back, empathy videos—and reports localized aberrations: the dorsal lateral prefrontal cortex (dlPFC) over-activates to solve incongruent Stroop trials, the right middle frontal gyrus fires harder when exhausted residents choose a diagnosis, or the anterior insula under-reacts while nurses watch another’s pain. The second camp acquires resting-state scans, builds whole-brain graphs, and notes that rich-club hubs weaken, characteristic path length rises, visual and limbic gradients warp, and long-range edges decay. Both camps are describing the same elephant from different angles. However, because their methodologies, statistics, and vocabularies differ, the field still lacks an integrated theory that explains how a sluggish global network produces the spot hyper- or hypo-activations observed during demanding tasks.

The pieces of the puzzle suggest a plausible sequence. Chronic overload appears to erode global integration first: longitudinal nurse data show that rich-club, feeder, and local connections all lose strength, with mid- and long-range links hit hardest. Graph metrics shift toward a more regular, lattice-like topology-efficient for local chatter but costly for cross-system coordination. Against this degraded infrastructure, the brain tries to meet workplace demands by cranking up regional effort. Hence, the dlPFC burns extra oxygen to keep attention steady, the middle frontal gyrus lights up to compensate for depleted illness scripts, and the caudate, when it still has volume, helps drive compensatory mental fatigue. The result is preserved accuracy at the price of neural inefficiency and subjective exhaustion.

Evidence for this compensatory model can already be glimpsed. In exhaustion disorder, smaller caudate volumes predict higher self-rated fatigue but paradoxically better working-memory scores, implying that patients marshal extra effort to mask structural loss. During repetitive psychosocial stress, healthy controls gradually dampen dACC activity. In contrast, burned-out workers ramp it up—as if they have no metabolic spare tire and must keep the pedal floored to stay on task. Yet such inferences are made from coarse averages: block-design BOLD contrasts and static graph measures that flatten second-by-second dynamics.

What is missing are bridging experiments that record both network health and moment-to-moment control demands in the same session. Imagine pairing event-related fMRI or fast TR multiband scans with pupillometry, heart-rate variability, and trial-level response times. Hidden-Markov or co-activation-pattern analyses could reveal whether burnout brains slip prematurely into high-cost “control-locked” states, linger there, or fail to re-enter low-load default-mode states between trials. Network control theory—quantifying the energy cost of moving from one connectivity state to another—could link macro-efficiency with micro-effort: a rising energetic bill would predict larger frontal BOLD bursts and steeper subjective fatigue.

Bridging also demands ecological validation. In-scanner tasks are brief and discrete, whereas real-world clinical shifts require sustained attention for hours. Deploying portable fNIRS or EEG during actual ward rounds, synced with workload logs and near-miss error reports, would test whether the lab-observed inefficiencies forecast lapses that matter. Wearables capturing sleep, heart-rate variability, and cortical hemodynamics could feed machine-learning models that predict when a resident’s network integration will dip below a critical threshold, enabling just-in-time breaks or support.

Finally, future studies must treat network integrity as a modifiable endpoint. Randomized interventions—sleep-banking schedules, structured exercise, mindfulness-based stress reduction—should include pre-/post-resting-state graphs and task-evoked efficiency metrics. If rich-club strength rebounds and frontal hyper-activation subsides in tandem with symptom relief, causal arrows sharpen; if symptoms improve while the network remains frayed, then compensatory mechanisms rather than structural repair may drive recovery. Either outcome informs treatment: rebuild the roads or teach drivers to coast.

Until such integrative designs are run, functional findings will sit in silos—local peaks here, global valleys there—and clinicians will struggle to translate them into actionable insight. Bridging the camps promises a unified map in which macro-scale disconnection and micro-scale over-exertion are two sides of one process, charting how the burned-out brain works harder, connects less, and ultimately tires out long before the shift ends.

### 7.5. Clinical Translation and Individual Prediction

Despite the intellectual appeal of mapping burnout’s neural circuitry, the ultimate test of the science is whether it improves real-world care, who is flagged for preventive action, which intervention is chosen, when it is delivered, and how progress is tracked. At present, neuroimaging sits on the periphery of clinical decision-making. No hospital or occupational-health team uses cortical-thickness scores or resting-state graph metrics to determine whether a nurse should come off night shifts, a resident needs structured mentoring, or a returning employee is genuinely ready for complete duties. The gap persists because most studies stop at group differences: the burned out cohort versus the control cohort. Clinicians, however, encounter individuals, and they need predictive tools that work one brain at a time.

Bridging that gap begins with diagnostics. Questionnaires such as the Maslach Burnout Inventory capture subjective distress but do not distinguish transient fatigue from looming collapse, nor can they differentiate burnout from overlapping syndromes like depression or chronic fatigue. Early evidence hints that imaging can add incremental value: amygdala hypertrophy appears in women who later develop affective symptoms; rapid erosion of rich-club efficiency precedes measurable exhaustion in trainee nurses; hyper-frontality during attention tasks predicts slower learning curves in medical residents. Yet none of these markers have been prospectively validated. The field needs multicentre cohorts in which baseline imaging, genetics, wearables, and workplace metrics feed into Bayesian or machine-learning models that are then frozen and tested on recruits-ideally predicting objective outcomes such as sick-leave days, medication starts, or logged clinical errors. Without that prospective “lockbox” phase, promising biomarkers will remain academic curiosities.

Treatment stratification presents a second translational frontier. Cognitive-behavioural therapy, mindfulness programmes, graded exercise, duty-hour limits, and organisational reforms help subsets of workers, but matching the right person to the proper intervention remains guesswork. Neuroimaging could supply a decision rule: workers whose ACC–amygdala connectivity fails to rebound during a brief neurofeedback test might benefit most from frontal-targeted transcranial direct-current stimulation, whereas those showing early striatal shrinkage and compensatory frontal hyper-activation might gain more from motivational interviewing plus workload redistribution. Testing such rules requires adaptive randomisation trials in which imaging phenotypes determine treatment allocation, followed by cost-effectiveness analyses that factor in scanner time against reduced attrition and malpractice risk.

Prognostication and relapse surveillance form the third pillar. Longitudinal data show that prefrontal thickness and caudate volume can recover while amygdala enlargement lingers; rich-club efficiency may normalise earlier than subjective energy. Embedding brief resting-state scans or portable neuro-cognitive probes at return-to-work visits could spot smouldering neural deficits that questionnaires miss, prompting gradual rather than abrupt resumption of full cognitive function. Mobile EEG or fNIRS headbands, calibrated to each employee’s MRI baseline, could deliver real-time cognitive-load readouts during shifts. When network-efficiency metrics drift past an individually derived threshold—say, two standard deviations below that worker’s rested baseline—the system could cue a micro-break, a peer check-in, or automated mindfulness prompts. Such closed-loop occupational-health ecosystems are technologically feasible; what is missing is the initial calibration data and the regulatory pathway linking neural alerts to staffing decisions.

Finally, neural metrics could become endpoints in interventional research, shortening trials and clarifying mechanisms. If an eight-week aerobic exercise regimen restores vmPFC thickness and dampens dACC over-reactivity before self-report scores budge, investigators gain an early signal to persevere; if biomarkers stay flat while symptoms improve, theory must shift from structural repair to psychological reframing or network compensation. For regulators and payers, objective neural change offers reassurance that benefits are biologically grounded, not placebo artifacts.

Taking burnout imaging from bench to bedside will demand prospective prediction models, phenotype-guided treatment algorithms, real-time monitoring platforms, and biomarker-anchored trials—all built on large, harmonised datasets and tested in pragmatic, frontline settings. Only then will the elegant maps of exhausted brains translate into fewer exhausted people.

## 8. Conclusions

The 17 MRI studies synthesised in this mechanistic review converge on a coherent neural signature of occupational burnout. Structurally, chronic work stress enlarges the amygdala—predominantly in women—and erodes striatal (caudate/putamen) and prefrontal grey matter, particularly in men; the hippocampus, by contrast, remains strikingly intact. Functionally, the brain responds to the loss of long-range “rich-club” highways by driving executive hubs harder, producing the well-documented mix of frontal hyper-activation, limbic dys-regulation, and global network inefficiency that underlies fatigue, emotional volatility, and decision-making lapses at the bedside or workstation.

These alterations satisfy every conservative criterion for disease: they are objective, reproducible, and disabling at both the individual and systems levels. They also differentiate burnout from related conditions such as depression or PTSD, confirming that the syndrome deserves nosological recognition. Encouragingly, the abnormalities are not fixed scars. Mindfulness programmes, structured exercise, cognitive–behavioural therapy, neurofeedback, and connectivity-guided non-invasive brain stimulation have each been shown—sometimes within weeks—to thicken atrophied cortex, shrink an over-reactive amygdala, or restitch frayed connectomes, with parallel clinical gains in energy, mood, and cognitive control.

Yet the evidence base is still young and methodologically uneven. Sample sizes rarely top 40, cross-sectional snapshots dominate, women in nursing cohorts outnumber men in technical roles, and preprocessing pipelines vary widely, fueling contradictory findings and limiting causal inference. Therefore, the next wave of studies must be longitudinal, high-field, multimodal, and hormone-aware, tracking trainees, shift workers, and knowledge-economy staff from “day zero” through peak strain and (ideally) recovery, while harmonising acquisition and analysis across centres.

## Figures and Tables

**Table 1:** Studies included in the review.

Reference	Participants (Groups & n)	MRI Modality/Paradigm	Key Brain Regions & Direction of Effect	Burnout Dimension(s) Linked	Notable Moderators/Comments
Structural MRI					
[26]	Exhaustion-syndrome 58 (22 M/36 F) vs. 65 controls	Structural MRI–amygdala/hippocampus subfields	Females only: ↑ Basal, Lateral, Central amygdala nuclei & whole amygdala (bilateral)	Occupational stress (MBI-GS)	Sex-specific hypertrophy; no hippocampal change
[27]	Nurses 43 (32 F)	VBM (structural)	↑ Burnout → ↓ GM in bilateral vmPFC & Left Insula (EE); ↓ Left vmPFC & Thalamus (DP)	Emotional exhaustion, depersonalization	vmPFC central node for stress modulation
[28]	Exhaustion disorder 55 (High MF 30/Low-Mod 25)	Structural MRI	High mental fatigue → ↓ Caudate & Putamen volumes	Mental fatigue (CIS)	Caudate volume ↔ working-memory via fatigue (mediated)
[29]	Chronic stress 30 vs. Controls 68	VBM + manual volumetry	↓ ACC & bilateral MFG GM; ↓ Caudate & Putamen volumes; no hippocampal/amygdala change	Burnout severity (MBI-GS)	Frontostriatal atrophy correlates with perceived stress
[30]	Exhaustion syndrome 48 (↺ 25) vs. Controls 80 (↺ 19)	Structural MRI (baseline & 1.5-yr follow-up)	Baseline: ↓ PFC thickness, ↓ Caudate (♂), ↑ Amygdala (♀), ↓ STG; Follow-up: PFC & Caudate recover, Amygdala persists	Chronic stress	Pronounced sex differences; partial reversibility with rehab
[31]	Burnout 40 vs. Controls 40	Structural MRI	↓ mPFC thickness; ↑ Amygdala volume; ↓ Caudate volume	Perceived stress/exhaustion	mPFC thinning ages faster under stress
[34]	ED women 300	Structural MRI–LPFC thickness	Small positive relation: stress ↑ ↔ thicker LPFC; LPFC unrelated to cognitive fatigue	Perceived stress; cognitive weariness	LPFC thickness neither mediates nor moderates stress–fatigue link
[39]	Exhaustion women 20 vs. Controls 16	Structural MRI–Hippocampal volumetry	No hippocampal change; cognitive deficits linked to blunted ACTH (not cortisol) & ↑ IL-1β	Burnout	Personality (↑ Harm avoidance) moderates vulnerability
Functional MRI					
[32]	Burnout 55 vs. Healthy 61	Task-fMRI–ScanSTRESS	No mean activation differences; dACC: BO ↑, HC ↓ across time (exposure-time effect)	Burnout severity	dACC “neuro-inflexibility” may precede clinical burnout
[25]	Physicians: Residents (10) vs. Faculty (17)	Task-fMRI–clinical reasoning (answer → read; reflect → read)	↑ Right MFG (BA 6) & PCC (BA 31) during answering ↓ Bilateral Precuneus (BA 7) & Right DLPFC (BA 9) during reflecting	Emotional exhaustion ↑ activity; depersonalization ↓ activity	Effects only in residents → greater cognitive load & inefficiency
[33]	Nurses (♀) 39 baseline → follow-up	Longitudinal rs-fMRI	Post-burnout: ↓ Rich-club, Feeder, Local connectivity (esp. mid/long range); weakened precuneus–basal-ganglia links	Emotional exhaustion; anxiety	Connectivity decline tracks symptom progression
[35]	Nurses 25	Task-fMRI–Empathy for pain	Higher burnout → ↓ AI/IFG & TPJ activation to pain	Emotional exhaustion, depersonalization	Supports emotional-dissonance (not compassion-fatigue) model
[36]	Work-stress 40 vs. Controls 70	rs-fMRI + EMG	Burnout: weaker amygdala → mPFC/dlPFC connectivity; stronger amygdala → Insula/Cerebellum; impaired down-regulation of negative affect	MBI-GS	Connectivity with ACC predicts emotion-regulation success
[37]	Pediatric residents 28	Task-fMRI–Stroop	Burnout ↑ → ↑ Right MFG/DLPFC activation (incongruent > congruent)	EE + DP composite	Indicates reduced cognitive efficiency under burnout
[38]	Women: LTSL 10 vs. MDD 10 vs. Controls 10	Task-fMRI–2-back & CVMT	LTSL group: hypoactivation in Left VLPFC & Right DLPFC (2-back); flattened diurnal cortisol	Work-stress burnout	Distinct from MDD despite similar symptoms
[40]	Burnout 32 vs. Controls 30	rs-fMRI + Graph theory	Burnout: ↑ Path length, ↓ Global efficiency; nodal ↑ Cuneus/Occipital, ↓ ACC centrality; ↓ Effective connectivity visual → Right Hippocampus	Full MBI	Suggests impaired global integration & sensory-memory loops
[41]	Female nurses 33 vs. 32	rs-fMRI + Gradient mapping + Transcriptomics	Burnout: distorted functional gradients (↑ eccentricity) in somatomotor & visual networks; linked to genes for circadian rhythm (↑) & synaptic function (↓)	EE & DP severity	Connectome changes map onto cell-type-specific gene sets

Abbreviations: ACC = anterior cingulate cortex; AI = anterior insula; dACC = dorsal ACC; DLPFC = dorsolateral prefrontal cortex; MFG = middle frontal gyrus; mPFC/vmPFC = (ventro)medial PFC; PCC = posterior cingulate cortex; EE = emotional exhaustion; DP = depersonalization; CIS = Checklist Individual Strength; LTSL = long-term sick leave; BA = Brodmann Area; MBI-GS = Maslach Burnout Inventory–General Survey; ACTH = Adrenocorticotropic Hormone; IL-1β = interleukin 1 beta; IFG = Inferior Frontal Gyrus; TPJ = Temporo-Parietal Junction; VBM = Voxel-Based Morphometry.

## Data Availability

No new data were created or analyzed in this study. Data sharing does not apply to this article.
